# Small Extracellular Vesicles Are Key Regulators of Non-cell Autonomous Intercellular Communication in Senescence via the Interferon Protein IFITM3

**DOI:** 10.1016/j.celrep.2019.05.095

**Published:** 2019-06-25

**Authors:** Michela Borghesan, Juan Fafián-Labora, Olga Eleftheriadou, Paula Carpintero-Fernández, Marta Paez-Ribes, Gema Vizcay-Barrena, Avital Swisa, Dror Kolodkin-Gal, Pilar Ximénez-Embún, Robert Lowe, Belen Martín-Martín, Hector Peinado, Javier Muñoz, Roland A. Fleck, Yuval Dor, Ittai Ben-Porath, Anna Vossenkamper, Daniel Muñoz-Espin, Ana O’Loghlen

**Affiliations:** 1 Epigenetics & Cellular Senescence Group, Blizard Institute, Barts and the London School of Medicine and Dentistry, Queen Mary University of London, 4 Newark Street, London E1 2AT, UK; 2 CRUK Cambridge Centre Early Detection Programme, Department of Oncology, Hutchison/MRC Research Centre, University of Cambridge, Cambridge CB2 0XZ, UK; 3 Centre for Ultrastructure Imaging, King’s College London, London SE1 1UL, UK; 4 Department of Developmental Biology and Cancer Research, Institute for Medical Research-Israel-Canada, Hebrew University-Hadassah Medical School, Jerusalem, Israel; 5 Proteomics Unit, Biotechnology Programme, Spanish National Cancer Research Centre (CNIO), Madrid 28029, Spain; 6 ProteoRed-ISCIII, Autonomous University of Madrid Campus, Cantoblanco, Madrid 28049, Spain; 7 Centre for Genomics and Child Health, Blizard Institute, Barts and the London School of Medicine and Dentistry, Queen Mary University of London, London E1 2AT, UK; 8 Microenvironment and Metastasis Group, Department of Molecular Oncology, Spanish National Cancer Research Center (CNIO), Madrid 28029, Spain; 9 Centre for Immunobiology, Blizard Institute, Barts and the London School of Medicine and Dentistry, Queen Mary University of London, London E1 2AT, UK

**Keywords:** exosomes, small extracellular vesicles, EV, paracrine senescence, OIS, DDIS, aging, interferon, IFITM3, fragilis

## Abstract

Senescence is a cellular phenotype present in health and disease, characterized by a stable cell-cycle arrest and an inflammatory response called senescence-associated secretory phenotype (SASP). The SASP is important in influencing the behavior of neighboring cells and altering the microenvironment; yet, this role has been mainly attributed to soluble factors. Here, we show that both the soluble factors and small extracellular vesicles (sEVs) are capable of transmitting paracrine senescence to nearby cells. Analysis of individual cells internalizing sEVs, using a Cre-reporter system, show a positive correlation between sEV uptake and senescence activation. We find an increase in the number of multivesicular bodies during senescence *in vivo*. sEV protein characterization by mass spectrometry (MS) followed by a functional siRNA screen identify interferon-induced transmembrane protein 3 (IFITM3) as being partially responsible for transmitting senescence to normal cells. We find that sEVs contribute to paracrine senescence.

## Highlights

• Small extracellular vesicles (sEVs) mediate paracrine senescence
• Inhibition of sEV biogenesis prevents paracrine senescence
• Multivesicular bodies and CD63 are increased during senescence *in vivo*
• MS analysis identifies IFITM3 as partially responsible for sEV-paracrine senescence

## Graphical Abstract

**Figure:**
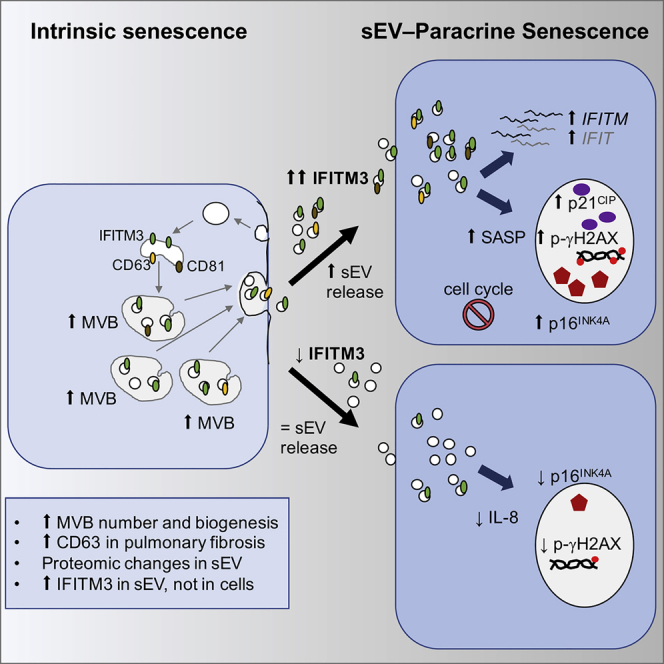

## Teaser

Borghesan et al. show that the soluble fraction and small extracellular vesicles (sEVs) mediate paracrine senescence. RNA sequencing and *loxP* reporter systems confirm sEV-mediated paracrine senescence, while preventing sEV release averts senescence. Mass spectrometry and functional analysis show that the IFN protein, IFITM3, is partially responsible for this phenotype.

## Introduction

The establishment of cellular senescence is categorized by a stable cell-cycle arrest and the capacity to modify the microenvironment through a particular secretome called SASP (senescence-associated secretory phenotype). The activation of senescence is a response to different cellular stresses to prevent the propagation of damaged cells and has been shown to occur *in vitro* and *in vivo*. In fact, an enrichment in the number of senescent cells has been observed *in vivo* during both biological and pathological processes such as development, cancer, fibrosis, and wound healing (He and Sharpless, 2017, Muñoz-Espín and Serrano, 2014). The SASP controls its surroundings by reinforcing senescence in an autocrine (cell autonomous) and paracrine (non-cell autonomous) manner, by recruiting immune cells to eliminate senescent cells and by inducing a stem cell-like phenotype in damaged cells (Mosteiro et al., 2016, Ocampo et al., 2016). The SASP provides the necessary balance to restore tissue homeostasis when it has been compromised. Paradoxically, the SASP can also contribute to the enhancement of tissue damage and the induction of inflammation and cancer proliferation. Overall, the mechanisms behind the pleiotropic activities of the SASP in different contexts are not well understood (Salama et al., 2014).

Most studies *in vitro* and *in vivo* have attributed the diverse functions of the SASP to individual protein components such as interleukin-6 (IL-6) or IL-8 to reinforce autocrine senescence (Acosta et al., 2008, Kuilman et al., 2008) or transforming growth factor β (TGF-β) as the main mediator of paracrine senescence (Acosta et al., 2013, Rapisarda et al., 2017) or to a dynamic SASP with a switch between TGF-β and IL-6 as predominant individual components (Hoare et al., 2016). However, it is still unclear how these diverse SASP components regulate senescence. In fact, inhibition of the SASP by blocking the mammalian target of rapamycin (mTOR) only partially prevents paracrine senescence, suggesting that alternative mechanisms may exist (Herranz et al., 2015, Laberge et al., 2015).

Exosomes are small extracellular vesicles (sEVs) (30–120 nm) of endocytic origin, whereas microvesicles are formed by the shedding of the plasma membrane. Exosomes and microvesicles are secreted by all cell types and found in most bodily fluids. Both contain nucleic acids, proteins, and lipids that generally reflect the status of the parental cell and can influence the behavior of recipient cells locally and systemically (O’Loghlen, 2018, Tkach and Théry, 2016). The increasing literature regarding EVs show that they are disease biomarkers (Melo et al., 2015), indicators of cancer metastasis (Hoshino et al., 2015), and therapeutic carriers (Kamerkar et al., 2017). However, although some studies have found an increase in the number of EVs released during senescence (Lehmann et al., 2008, Takasugi et al., 2017), very little is known regarding the role that EVs play as SASP mediators in the senescent microenvironment.

Here, we show that both the soluble and sEV fractions transmit paracrine senescence (called sEV-PS herein). The analysis of individual cells internalizing sEVs using a reporter system shows a positive correlation between the uptake of sEVs and paracrine senescence. We can also observe an increase in multivesicular body (MVB) formation in a mouse model of oncogene-induced senescence (OIS) and high CD63 staining in human lung fibrotic lesions enriched in senescent cells. sEV protein characterization by mass spectrometry (MS) followed by a functional small interfering RNA (siRNA) screen identify the interferon (IFN)-induced transmembrane protein 3 (IFITM3) within sEVs as partially responsible for transmitting senescence to normal cells.

## Results

### sEVs and Soluble Factors from Senescent Fibroblasts Mediate Paracrine Senescence

**Figure 1:**
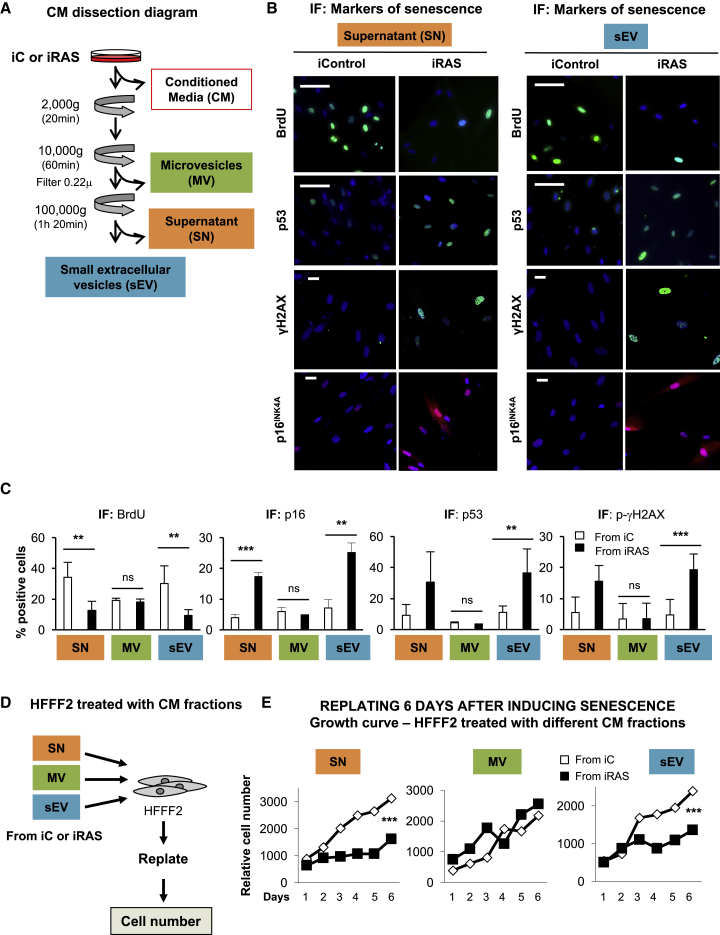
Small Extracellular Vesicles (sEVs) and Soluble Factors Form Part of the Senescent Secretome and Mediate Paracrine Senescence in Normal HFFF2s (A) Schematic representation of the proof-of-concept experiments performed to show that sEVs form part of the senescent secretome. HFFF2 human primary fibroblasts expressing a vector encoding an inducible form of H-RAS^G12V^ ER:RAS (iRAS) or an empty vector (iC) were treated with 200 nM 4OHT for 2 days and allowed to produce conditioned media (CM) for a further 3–5 days. This CM was taken from iC or iRAS HFFF2s and tested for the ability to induce senescence in HFFF2 as a whole (Figure S1A) or (Figures 1B–1E) processed by serial ultracentrifugation to evaluate the effect of the different fractions: supernatant (SN), large extracellular vesicles (MVs), or sEVs to induce paracrine senescence in HFFF2s. (B and C) HFFF2 fibroblasts were treated for 72 h with the different fractions of the CM (SN, MV, or sEV) from iC or iRAS cells, and the endogenous expression of different markers of senescence was determined as shown in (B) representative pictures and by (C) quantifying the percentage of cells staining positive for different antibodies by IF. The graphs represent the means ± SDs of 2–6 independent experiments. Scale bars: 100 μm for BrdU and p53 and 30 μm for p-γH2AX and p16^INK4A^. (D and E) HFFF2 cells were treated twice for 72 h with the different fractions of the CM, replated, and counted on different days. (D) Scheme of the experiments performed. (E) Growth curves showing the mean of 3 independent experiments. See also Figure S1.

To investigate whether EVs act as intercellular mediators during senescence, we took advantage of HFFF2 human foreskin primary fibroblasts expressing an empty vector or oncogenic H-RAS^G12V^ in a 4-hydroxytamoxifen (4OHT)-inducible form (ER:EV or ER:H-RAS^G12V^). These cells undergo senescence upon treatment with 200 nM 4OHT (Rapisarda et al., 2017), without activating the apoptosis pathway, and we have called them iC or iRAS, respectively. The treatment of HFFF2s with whole conditioned media (CM) from iC and iRAS (mimicking OIS) confirm previous findings that senescence can be transmitted in a non-cell-autonomous (paracrine) fashion (Acosta et al., 2013). Recipient HFFF2s present a decrease in cell proliferation (staining with crystal violet) and an increase in the percentage of cells staining positive for senescence-associated-β-galactosidase (SA-β-Gal) (Figure S1A). Next, we dissected the CM from iC and iRAS HFFF2s and isolated large (MV) and small EV (sEV) from the same CM. We followed the well-characterized serial ultracentrifugation protocol (Théry et al., 2006) filtering the sEV fraction with a 0.22-μm filter and compared the effect of the MV and sEV fractions in inducing paracrine senescence in comparison to the supernatant (SN) fraction (soluble fraction depleted of MV and sEV) (Figure 1A). Both the SN and sEV fraction from iRAS cells induced an upregulation of several markers of senescence in normal HFFF2 compared to iC, as shown by a decrease in bromodeoxyuridine (BrdU) incorporation, an increase in p16^INK4A^ protein expression levels, the percentage of positive cells for phosphorylated-γH2AX (p-γH2AX), and the accumulation of p53 by immunofluorescence (IF) (Figures 1B and 1C). Although MVs isolated from iC or iRAS induced a reduction in the incorporation of BrdU, no other markers of senescence were observed, suggesting that only the SN and sEVs from iRAS cells transmit paracrine senescence. Next, to determine whether the cell-cycle arrest observed by the delay in proliferation was maintained long term, we treated HFFF2 twice for 72 h with the different fractions, replated the cells, and determined the growth potential at different days (Figure 1D). As observed in Figure 1E, only the SN and sEV fractions derived from iRAS cells were able to induce a delay in proliferation in comparison with the iC. To confirm sEV-PS induction, we treated HFFF2s with increasing concentrations of sEVs derived from iRAS compared to the highest dose of sEVs from iC and observed a dose-dependent senescent response by quantifying the percentage of cells staining positive for SA-β-Gal and the levels of p53^+^ and BrdU^+^ cells by IF (Figures S1B and S1C). Furthermore, treatment of HFFF2 with the same number of sEVs also transmits the senescent phenotype as shown by the decrease in BrdU incorporation and IL-8 staining by IF (Figure S1D), suggesting that the sEV content but not particle number is responsible for sEV-PS. To confirm that sEV-PS was not due to contaminants present in our sEV preparations, we isolated sEVs using an alternative isolation technique, size exclusion chromatography (SEC). The absence of protein contaminants and presence of particles in the sEV fractions were determined (not shown). As can be seen in Figure S1E, sEV isolated by SEC can also mediate paracrine senescence. Therefore, both the soluble fraction and sEVs are responsible for mediating a delay in proliferation and inducing an increase in the expression levels of diverse biomarkers of senescence.

### The sEV Fraction Extracted from iRAS Cells Contains Exosome-like Particles

Next, we wanted to determine whether the sEV fraction isolated from iC and iRAS cells contained exosomes. The comparison of immunoblotting analysis of cells and sEV lysates derived from iC and iRAS cells showed the presence of different exosome-related proteins (CD63, TSG101, and ALIX) in our sEV preparations (Figures S1F and S1G). Furthermore, we confirmed the absence of proteins related to intracellular compartments, calnexin (endoplasmic reticulum), and COX IV (mitochondria) to assess the purity of our sEV preparations (Figure S1G). Changes in the expression levels of annexin V could not be observed between iC and iRAS cells lysates, while it was detected in sEVs derived from iRAS cells, as described (Ostrowski et al., 2010, Théry et al., 2018) (Figure S1H). We verified the sEV morphology and size by transmission electron microscopy (TEM) (Figure S1I) and the sEV population released in iRAS cells by specifically capturing sEVs onto beads coated with a CD63 antibody, followed by a CD81-phycoerythrin (PE)-conjugated antibody. In fact, at different time points after inducing senescence, an increase can be seen in CD81 fluorescence intensity and therefore a release in sEVs containing simultaneously CD63^+^/CD81^+^ (Figure S1J). Previous studies have shown that cells undergoing senescence release more sEVs (Kavanagh et al., 2017, Lehmann et al., 2008, Takasugi et al., 2017). Thus, we quantified the number and size of sEVs by nanoparticle tracking analysis (NTA) in iRAS cells and HFFF2s treated with 50 μM etoposide (Etop) for 48 h, which does not induce apoptosis, followed by 5 days’ incubation with fresh medium (mimicking DNA damage-induced senescence [DDIS]). As shown in Figure S1K, an increase in sEV release during senescence can be observed by NTA analysis. We also show an increase in the release of sEVs during senescence in a variety of human cells and mouse cells: (1) human breast primary fibroblasts expressing ER:H-RAS^G12V^ (Rapisarda et al., 2017); (2) breast cancer cells, MCF7, treated with 500 nM of the CDK4/6 inhibitor palbociclib (Palbo) for 10 days as previously described (Rapisarda et al., 2017); and (3) *ex vivo* mouse hepatic stellate cells (mHSCs) derived from an adult mouse harboring a doxycycline (Dox)-inducible construct to express shp53 (Krizhanovsky et al., 2008, Lujambio et al., 2013) (Figure S1L). We did not observe changes in sEV size distribution with any of the triggers of senescence (data not shown). Our results show that different triggers of senescence induce the release of sEVs in a variety of human cells and mouse cells and that some of these sEVs present exosome-like features.

### sEV Isolated from iRAS Induce Paracrine Senescence in HFFF2s

**Figure 2:**
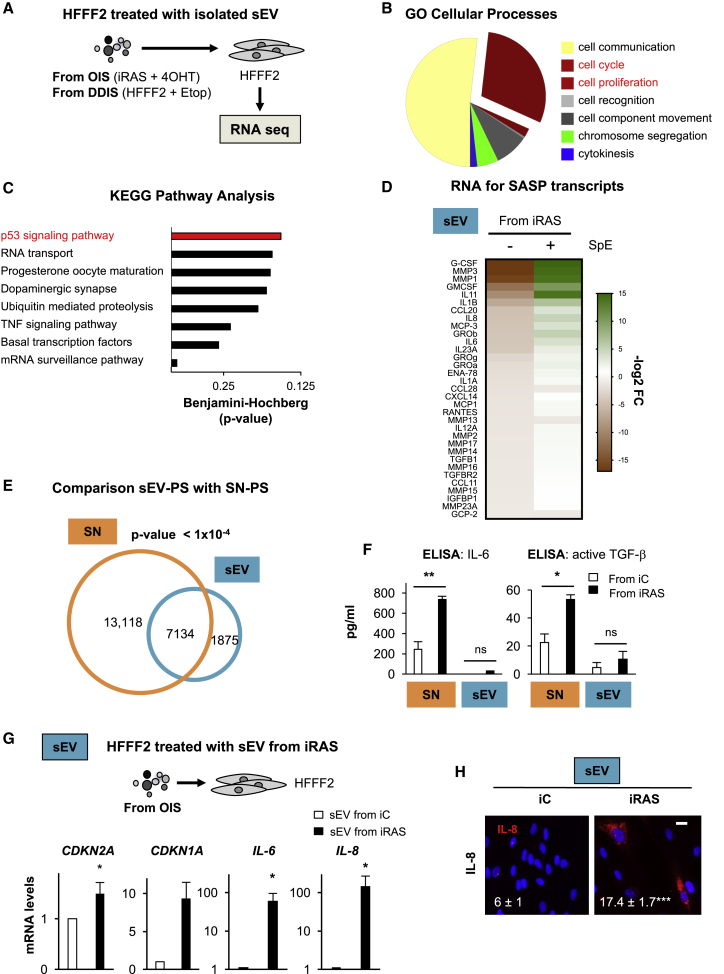
Transcriptome Analysis Shows that sEVs Induce a Senescent Signature (A) Schematic representation of the experimental setting where HFFF2s were treated for 72 h, with sEVs isolated from iRAS cells (mimicking OIS) or HFFF2s treated with Etop (mimicking DDIS) and sent for RNA sequencing. (B) Gene Ontology (GO) analysis for genes involved in cellular processes with >2 log_2_ fold differential expression and p < 0.05 in both OIS- and DDIS-treated HFFF2s. The pie chart shows a high proportion of genes related to the “cell-cycle” and “cell proliferation” pathways. (C) Kyoto Encyclopedia of Genes and Genomes (KEGG) pathway analysis shows the “p53 signaling pathway” as representative upon HFFF2 treatment with sEVs isolated from senescent cells (from both OIS and DDIS). (D) Bioinformatics analysis of SASP mRNA transcripts in HFFF2 treated with sEVs from iRAS and iC cells. The upregulation of SASP transcripts is prevented when iRAS cells were treated with 5 μM spiroepoxide (SpE; inhibitor of the enzyme neutral sphingomyelinase N-SMase). Data have been normalized to the control and represent the reads per kilobase million (RPKM)-log_2_ fold difference. (E) Comparison between the paracrine senescence (PS) signature identified by Acosta et al. (2013) by soluble factors (SN) with the sEV-PS signature. (F) ELISA to determine the concentration of IL-6 and active TGF-β present in the SN and sEV lysed fractions. (G and H) HFFF2 treated with sEVs derived from iRAS cells induce an upregulation of cell-cycle inhibitors (*CDKN2A*, *CDKN1A*) and SASP (*IL-6*, *IL-8*) at the mRNA level, as shown by qPCR analysis (G) and an increase in the percentage of cells staining positive for IL-8 by IF (H). Scale bar, 30 μm. All data represent means ± SDs of 2–4 experiments. See also Figure S2.

To confirm that sEVs from senescent cells induce paracrine senescence (sEV-PS), we performed RNA sequencing (RNA-seq) of HFFF2s treated with sEVs derived from iRAS and from HFFF2s treated with Etop, mimicking OIS and DDIS, respectively (Figure 2A). Gene Ontology (GO) analysis of genes deregulated by >2 log_2_ fold difference expression levels and p < 0.05 in both OIS- and DDIS-sEV-treated HFFF2s confirmed a shared “cell cycle” and “cell proliferation” signature (Figure 2B), with the “p53 signaling pathway” being overrepresented (Figure 2C). Furthermore, HFFF2 cells treated with sEVs derived from both OIS and DDIS also showed a significant SASP and “inflammatory response” signature (Figures 2D and S2A). We next used a small molecule inhibitor, spiroepoxide (SpE), which has been described as blocking the enzyme neutral sphingomyelinase (N-SMase) and as inhibiting exosome biogenesis and release (Hannun and Obeid, 2008, Trajkovic et al., 2008). Treatment of iRAS cells with SpE (5 μM) did not induce cell death. However, we did observe prevention of the senescent signature by RNA-seq and a decrease in sEV release by fluorescence-activated cell sorting (FACS) (Figures 2D and S2B) and NTA (Figure S2C). Comparison of the paracrine senescent signature previously described to be mediated by soluble factors (Acosta et al., 2013) (SN-PS) with the signature provided by sEVs (sEV-PS) in the RNA-seq data show a significant correlation between both mediators of senescence (Figure 2E). ELISA analysis of different SASP components in the SN or within lysed sEVs shows that the concentration of IL-6, IL-8, or active TGF-β (Figures 2F and S2D) in sEV lysates from iRAS is similar to or lower than the concentration observed in the SN from iC samples, which cannot induce senescence (Figure 1). Similar results were observed analyzing the CM (data not shown), suggesting that these individual SASP components are not responsible for sEV-PS.

Validation of the RNA-seq data shows that HFFF2s treated with iC and iRAS-derived sEVs show an increase in the mRNA expression levels of cell-cycle regulators *CDKN2A, CDKN1A*, components of the SASP, and integrin β3 subunit (*ITGB3*), which regulates senescence (Rapisarda et al., 2017) (Figures 2G and S2E). A comparable response was observed by IF and immunoblotting at the protein level (Figures 2H and S2F). Similar results were obtained in HFFF2s treated with sEVs isolated from HFFF2s undergoing DDIS (Figure S2G). iRAS HFFF2s expressing a vector encoding for 2 previously characterized short hairpin RNAs (shRNAs) targeting *TP53* (shp53) and *CDKN2A* (shp16) that prevent the establishment of senescence (Acosta et al., 2008, Rapisarda et al., 2017) show a reduction in the release of sEVs during senescence (Figure S2H). We next wanted to confirm that the sEV-PS response was not due to HFFF2 cells undergoing genotoxic stress. We isolated sEVs from HFFF2 cells treated with Etop for 1 day (pre-senescent) and 6 days (senescent) and treated normal HFFF2s to determine their response. As shown in Figures S2I and S1J, the 6-day-derived sEVs induced a reduction in BrdU incorporation and an increase in p-γ-H2AX, while no effect was observed upon treatment with the 1-day isolated sEVs. These data suggest that sEVs mediate paracrine senescence independently of the SASP and the induction of early genotoxic damage.

### Inhibition of N-SMase Enzyme Prevents Paracrine Senescence

**Figure 3:**
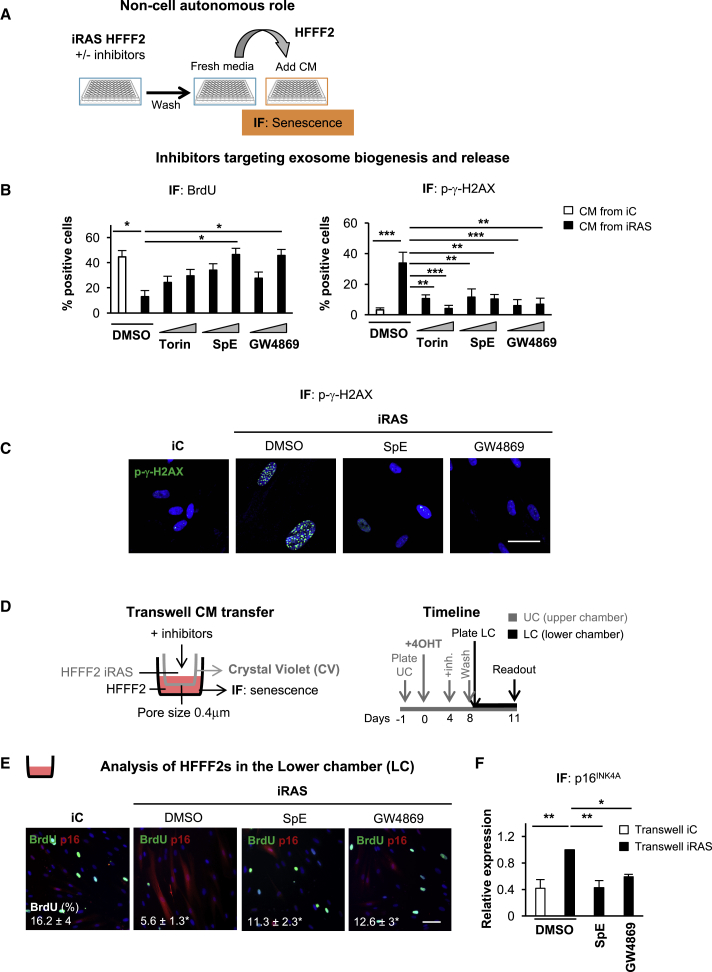
Inhibition of the Enzyme Neutral Sphingomyelinase, N-SMase, Prevents Paracrine Senescence (A) Schematic representation of the experimental settings to determine whether inhibition of N-SMase influences paracrine senescence. iRAS cells were treated with 200 nM 4OHT for 2 days, followed by treatment with different concentrations of Torin-2 (25 and 50 nM) or 2 independent N-SMase inhibitors: GW4869 (1 and 10 μM) and SpE (2 and 5 μM) for 3 days. After the incubation with the inhibitors, cells were washed and allowed to produce fresh CM for 72 h. Normal HFFF2s were then incubated with this CM for a further 72 h. (B) CM-treated HFFF2 fibroblasts were then stained to assess for the percentage of cells expressing markers of senescence: incorporation of BrdU and p-γH2AX by IF (means ± SEMs of 3–4 experiments; one-way ANOVA). (C) Representative pictures for p-γH2AX by IF of HFFF2s treated with the CM from iRAS with or without SpE or GW4869. Scale bar, 50 μm. (D) Schematic representation of the experimental settings and timings to test the implication of small EVs using the Transwell system with a membrane pore size of 0.4 μm. (E and F) The lower chamber was stained to quantify the percentage of cells incorporating BrdU and expressing p16^INK4A^ by IF. Representative pictures and the quantification of BrdU incorporation (E) and p16^INK4A^ (F) are shown. Scale bar, 100 μm. One-way ANOVA test was performed. All data show the means ± SEMs of 2–3 independent experiments. See also Figure S3.

Next, we investigated whether inhibition of the N-SMase enzyme prevents sEV-PS. For this, we treated iRAS cells with increasing concentrations of 2 independent N-SMase inhibitors: SpE (2 and 5 μM) and GW4869 (1 and 10 μM). Torin-2 (25 and 50 nM), an mTOR inhibitor that suppresses the SASP (Herranz et al., 2015, Laberge et al., 2015), was used as a control. None of the inhibitors or concentrations used induced cellular toxicity. We then washed the treated cells, added fresh media for 72 h, and treated normal HFFF2s for an additional 72 h with the altered CMs (Figures 3A–3C). As shown in Figure 3B, the highest concentrations of SpE and GW4869 prevented the cell-cycle arrest mediated by the CM of iRAS, while Torin-2 had no effect (Herranz et al., 2015, Laberge et al., 2015). The bypass of the arrest mediated by the CM from iRAS cells treated with SpE correlated with a decrease in the expression levels of p21^CIP^ (Figure S3A). Furthermore, the CM from both GW4869- and SpE-treated cells prevented the upregulation of IL-8 and p-γH2AX mediated by the CM of iRAS cells in normal HFFF2 (Figures 3B, 3C, S3B, and S3C). A similar response was observed in an additional strain of fibroblasts, IMR-90, by measuring the levels of expression of p21^CIP^ and p-γH2AX by IF (Figures S3D and S3E). Next, we used Transwell inserts to induce a physical separation between the cells and used a 0.4-μm pore membrane to avoid the transfer of larger vesicles. We plated iC and iRAS with the different treatments in the upper chamber (UC) and later plated HFFF2 in the lower chamber (LC), adding fresh CM (Figure 3D). As per our previous data, treatment with GW4869 and SpE prevented paracrine senescence, which was observed by an increase in cells incorporating BrdU in the LC and expressing lower levels of p16^INK4A^ (Figures 3E and 3F). These data suggest that the dose-dependent inhibition of N-SMase using two independent small-molecule inhibitors prevents paracrine senescence.

### The Endosome Pathway Is Enhanced during Senescence *In Vivo*

**Figure 4:**
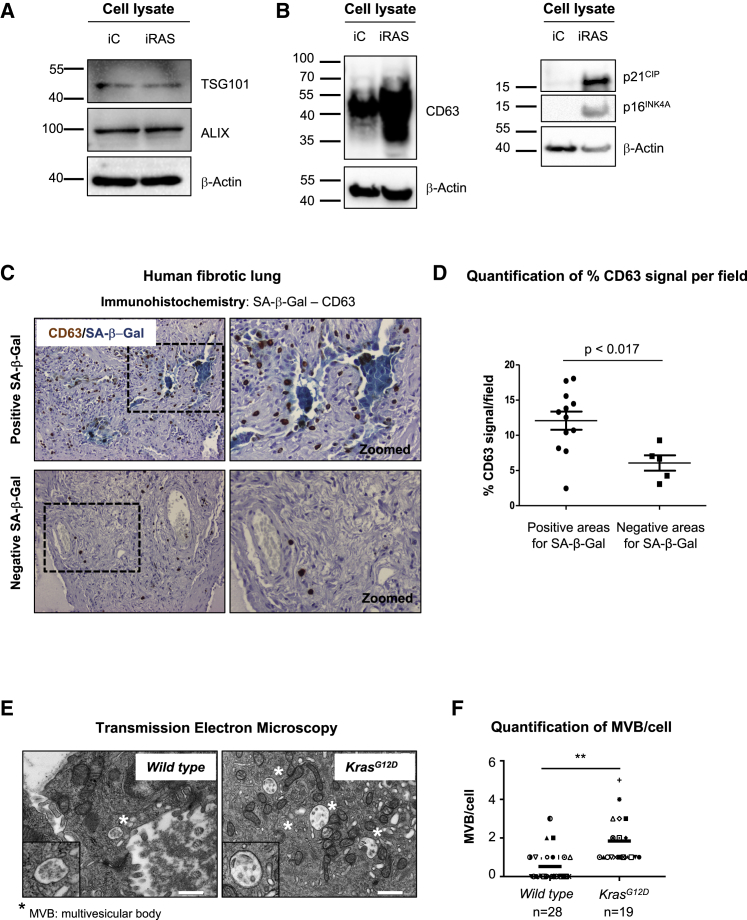
Increase in CD63 Staining and Multivesicular Body Formation during Senescence *In Vivo* (A and B) Immunoblot for endogenous expression of (A) ALIX and TSG101 and (B) CD63. p21^CIP^ and p16^INK4A^ upregulation are positive controls to confirm the induction of senescence. β-Actin represents the loading control. (C) Immunohistochemistry for SA-β-Gal (blue staining) and CD63 (brown signal) in a representative human sample of lung fibrosis. H&E staining is shown (violet). Pictures at top represent areas enriched in SA-β-Gal^+^ cells, and pictures at bottom show areas with low SA-β-Gal^+^ cells. (D) Quantification of positive pixels for CD63 per field, normalized by the H&E staining. The Mann-Whitney test was performed. (E) Representative transmission electron microscopy images of multivesicular bodies (MVBs) in *wild-type* (*WT*) and *Kras*^*G12D*^-derived PanIN (*Kras*^*G12D*^). Scale bar, 500 nm. (F) Quantification of MVB per cell in *WT* (n = 28 cells) and PanINs (n = 19 cells). See also Figure S4.

We next investigated whether proteins involved in the endosome pathway were differentially expressed during senescence. HFFF2 cell lysates extracted from iC and iRAS cells showed no differences at the endogenous levels of TSG101 or ALIX during senescence, while a clear increase in CD63 and other markers of senescence (p16^INK4A^ and p21^CIP^) were detected (Figures 4A and 4B). Subsequently, to test whether CD63 expression was increased *in vivo*, we took advantage of human fibrotic lung samples previously described to be enriched in senescent cells (Schafer et al., 2017) and evaluated the expression levels of CD63 by immunohistochemistry (IHC). We observed a significant increase in CD63 signal in areas enriched for SA-β-Gal^+^ cells in comparison with areas with fewer SA-β-Gal^+^ cells in a variety of human fibrotic lung samples (Figures 4C, 4D, and S4A). Next, to determine whether changes in the endocytic pathway occur during senescence *in vivo*, we took advantage of a mouse model of OIS. We used a transgenic mouse expressing a conditional *Ptf1a*^*Cre*^-driven activated *Kras* (*Ptf1a*^*Cre*^*;lsl-Kras*^*G12D*^) allele, which develops premalignant intra-epithelial neoplasias (PanIN) (Hingorani et al., 2003), and presents different markers and features of senescence (Figure S4B) (Caldwell et al., 2012, Morton et al., 2010). We subjected the pancreas from *wild-type* (*WT*) or *Kras*^*G12D*^ mice to electron microscopy staining and imaging and found that *Kras*^*G12D*^-derived PanIN showed an increase in MVB formation in comparison with *WT* epithelial ducts (Figures 4E and 4F). We also observed an increase in the number of lysosomes, secretory vesicles, and mitochondria per cell in PanINs when compared to *WT* epithelial ducts (Figure S4B) (Helman et al., 2016). Therefore, we observed that the endocytic pathway is altered during senescence in humans and mice *in vivo*.

### sEVs from iC and iRAS Fibroblasts Are Internalized by Normal HFFF2s

**Figure 5:**
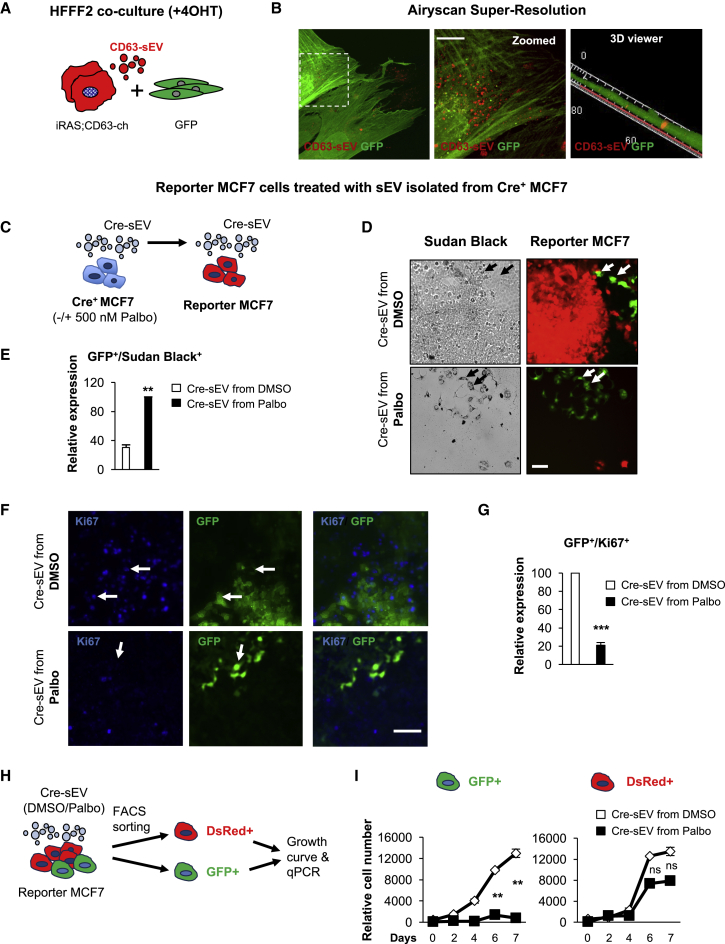
The Uptake of sEVs Derived from Cells Undergoing Senescence Induces Paracrine Senescence (A) Schematic representation of HFFF2 fibroblasts used for the co-culture experiments. Co-culture of HFFF2 expressing a GFP plasmid and iRAS HFFF2 fibroblasts expressing a retroviral construct encoding for mCherry-CD63 (iRAS;CD63-ch). Cells were plated in a 1:1 ratio and treated with 4OHT for 48 h, followed by 3–4 days with fresh media. (B) Representative images showing the uptake of CD63-cherry^+^ sEVs (CD63-sEV) in GFP cells acquired with the super-resolution microscope Airyscan. Right, a 3D reconstruction of confocal z stack images showing CD63-sEVs inside GFP cells. Scale bar, 20 μm. (C) MCF7 breast cancer cells expressing a Cre recombinase construct (Cre^+^ MCF7) were treated with DMSO or 500 nM palbociclib (Palbo) for 10 days to induce senescence. sEVs were purified from Cre^+^ MCF7 cells (Cre-sEV) and used to treat MCF7 cells expressing a fluorescent reporter gene, which switches from expressing DsRed to eGFP upon sEV internalization (reporter MCF7). (D) Representative pictures showing sEV uptake (GFP^+^ cells) in reporter MCF7s treated with sEVs isolated from Cre^+^ MCF7 treated with DMSO or Palbo. GFP^+^ cells are also Sudan Black^+^. (E) Quantification of the percentage of GFP^+^ reporter MCF7 treated with sEVs presenting with Sudan Black staining. (F) Pictures display sEV uptake (GFP^+^) in reporter MCF7 cells incubated with Cre-sEVs from DMSO- or Palbo-treated cells. Arrows show that GFP^+^ cells treated with Cre-sEV from DMSO cells are also positive for Ki67, while GFP^+^ cells incubated with Cre-sEV from Palbo-treated cells are negative for Ki67. (G) Quantification of the percentage of GFP^+^/Ki67^+^ reporter MCF7 treated with sEVs purified from DMSO- or Palbo-treated Cre^+^ MCF7. (D and F) Scale bar, 100 μm. (H and I) GFP^+^ and DsRed^+^ MCF7 treated with sEVs from both DMSO and Palbo cells were sorted by FACS. (H) Scheme of the experimental settings. (I) Growth curve showing the GFP^+^ and DsRed^+^ MCF7 populations. See also Figure S5.

Next, we investigated whether sEVs from both iC and iRAS cells were being internalized by HFFF2. For this, we generated iRAS HFFF2 cells expressing an mCherry-CD63 construct (iRAS;CD63-ch), which release mCherry^+^ sEVs (CD63-sEV), and HFFF2s expressing GFP. First, we co-cultured both GFP^+^ and iRAS;CD63-ch^+^ cells in a 1:1 ratio with 200 nM 4OHT for 2 days, followed by replenishing with fresh media for 4 days (Figure 5A); after this, the cells were washed twice with PBS to wash away all non-internalized EVs. We confirmed the presence of CD63-sEV transfer in GFP^+^ HFFF2s by confocal microscopy (Figure S5A) and the super-resolution Airyscan microscope (Figure 5B). Furthermore, 3D rotation of a z stack image (Figure 5B, right panel) and a 3D modeling video (Video S1) show internalized CD63-sEVs within GFP^+^ HFFF2s. To avoid confounding effects of the SASP, we purified CD63-sEVs from iRAS;CD63-ch and iC;CD63-ch cells and treated normal unlabeled HFFF2 cells (Figure S5B). Overall, we observed that HFFF2 did internalize CD63-sEV particles.

Video S1. Internalization of CD63-sEV within GFP^+^ HFFF2s, Related to Figure 5 A 3D modeling video shows that some sEV positive for CD63 can be observed in both the bottom and upper section of the GFP^+^ HFFF2 incubated with CD63-sEV.

### Cre-*loxP* Reporter System Shows a Positive Correlation between sEV Uptake and Induction of Paracrine Senescence

To further confirm a role for sEV-PS and sEV internalization, we used the Cre *loxP* reporter system described by Zomer et al., 2015, Zomer et al., 2016). For this, we generated MCF7 breast cancer cells expressing Cre recombinase (Cre^+^ MCF7) and MCF7 expressing a *loxP* flanked DsRed construct (reporter MCF7), which when recombined allows the expression of a downstream GFP gene (Figures 5C and S5C). The percentage of recombination was between 1% and 2%, as previously described (Zomer et al., 2015), while no spontaneous recombination was observed. As previously shown with HFFF2s, MCF7 incubated with sEVs isolated from Palbo-treated MCF7 undergoes paracrine senescence. By measuring the percentage of GFP^+^ reporter MCF7 cells (that have taken up Cre-sEV), we observed a positive correlation between GFP^+^ and Sudan Black^+^ cells (Figures 5D and 5E). In fact, the percentage of GFP^+^/Sudan Black^+^ cells is higher upon treatment with Cre-sEVs isolated from Palbo-treated MCF7 (Figure 5E). In addition, we observed that GFP^+^ MCF7 cells were negative for Ki67 when treated with Cre-sEVs derived from Palbo-treated cells (Figure 5F) and that the percentage of GFP^+^/Ki67^+^ MCF7 decreased upon treatment with Palbo-derived Cre-sEVs (Figure 5G). Upregulation of the mRNA levels of *IL-6, IL-8*, and *CDKN1A* (Figure S5E), in addition to an increase in cells staining positive for Sudan Black and a decrease in cell number by DAPI, can be observed in Cre-sEV Palbo-treated reporter MCF7 cells (Figure S5F). No differences were detected in the uptake of reporter MCF7 incubated with DMSO or Palbo-derived sEVs (data not shown). Next, we sorted by FACS the GFP^+^ and DsRed^+^ MCF7 population treated with DMSO or Palbo-isolated sEVs and evaluated the differences in the induction of senescence (Figure 5H). Our data show that the Palbo-treated sEV GFP^+^ population presents reduced proliferation and upregulates several markers of senescence, in contrast to the DsRed^+^ cell population (Figures 5I, S5G, and S5H). Our data show a positive correlation between the uptake of sEVs derived from Palbo-treated MCF7 cells and the establishment of sEV-PS.

### The sEV Protein Content Derived from Control and Senescent Cells Is Diverse

**Figure 6:**
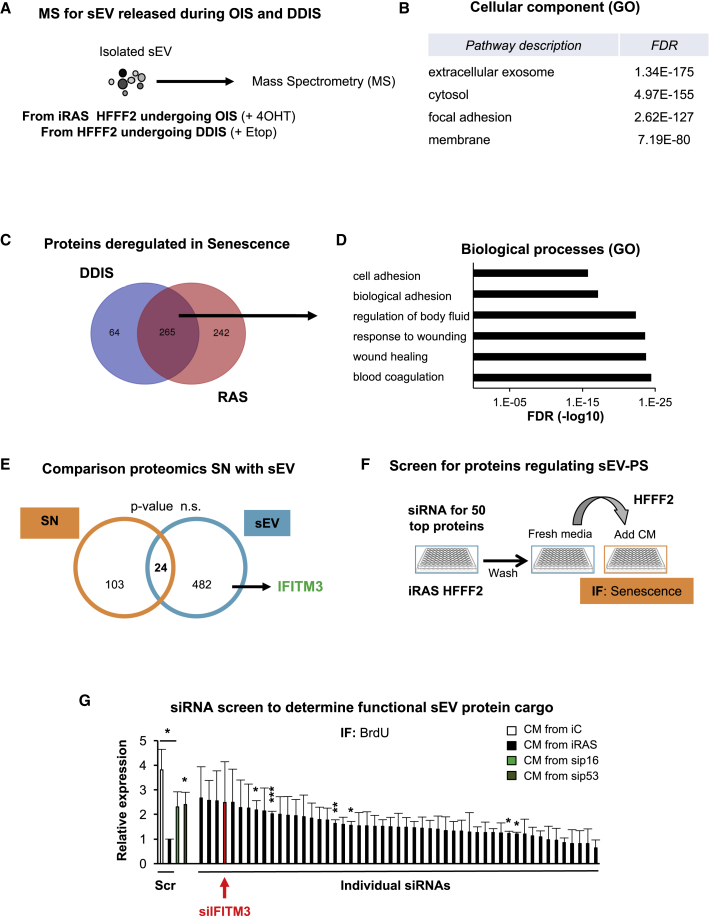
MS Proteomic Analysis Reveals a Specific Cargo in sEVs Derived from Senescent Cells (A) Scheme showing the mass spectrometry (MS) approach. sEVs were isolated from HFFF2 undergoing either OIS or DDIS from 2 independent experiments and were sent for label-free MS analysis. (B) DAVID GO analysis for the 1,600 proteins detected by the MS group into the “extracellular exosome” pathway. FDR, false discovery rate. (C) Venn diagram for proteins with >2 log_2_ differential expression and <0.01 FDR in sEVs released during OIS and DDIS compared to controls shows 265 common proteins that are deregulated during senescence. (D) GO analysis groups the 265 proteins into biological processes related to senescence-like “wound healing” and “response to wound healing.” (E) Comparison of the components of the soluble factors (SN) reported by Acosta et al. (2013) and the protein composition found within sEVs during senescence. IFITM3 is an example of a protein found specifically in the sEV fraction during senescence. (F) Schematic diagram showing the strategy used for the performance of the siRNA screen. Briefly, after transfection with the siRNA, the whole CM was washed out and replenished with fresh media for 72 h. (G) Screen using SMARTpool siRNA targeting the 50 most upregulated proteins in both OIS and DDIS with >4 peptide fold difference between control and senescence. Data show BrdU staining by IF for HFFF2s treated with different CM. A scramble siRNA (Scr) and siRNA targeting *TP53* (sip53) and *CDKN2A* (sip16) were used as negative and positive controls (green bars). siIFITM3 is highlighted in red. Data represent the means ± SEMs of 3 independent experiments. See also Figure S6.

Based on our previous findings that an equal number of control and senescent-derived sEVs induce sEV-PS and that sEV-PS is concentration dependent, we hypothesized that the sEV protein content may differ in senescence. For this, we subjected isolated sEVs from iRAS and Etop-treated HFFF2 and control cells to label-free quantitative MS analysis (Figure 6A). GO analysis of the 1,600 proteins identified show the cellular component “extracellular exosome” is overrepresented (Figure 6B). To identify proteins common to both stimuli inducing senescence, we selected proteins deregulated in both OIS- and DDIS-sEV versus control sEV with >2 log_2_ fold change difference and adjusted false discovery rate (FDR) <0.01 in both OIS and DDIS. The Venn diagram analysis shows that 265 proteins are deregulated in sEVs in both OIS and DDIS (Figure 6C), which group into GO biological processes related to senescence as “wound healing” and “cell adhesion” (Figure 6D). Volcano plot analysis comparing individually OIS- and DDIS-sEV protein content versus their respective controls shows that most proteins identified within sEVs derived from senescent HFFF2 are upregulated (Figure S6A). We found few previously described components of the SASP within the sEV proteomics analysis. A comparative analysis between the soluble factors found in previous publications (Acosta et al., 2013) and the sEV protein content show no significant correlation (Figure 6E). Therefore, the protein content in sEVs derived from cells undergoing DDIS and OIS is different from control-derived sEV protein content, although many sEV proteins are common to both senescence inducers.

To establish which of these proteins common to OIS- and DDIS-derived sEVs are essential to mediate paracrine senescence, we determined an additional cutoff, as follows: (1) >2 log_2_ fold change, (2) p < 0.05, and (3) >4 peptide fold change between control and senescent sEVs. After applying this cutoff, we selected the top 50 most abundant proteins upregulated in both DDIS and OIS (Figure 6F; Table S1) and conducted a small-scale screen using siRNA SMARTpool. We used the whole CM from iRAS as an indicator to determine overall paracrine senescence in normal HFFF2 using a scramble (Scr) siRNA or previously validated siRNA targeting *TP53* (sip53) and *CDKN2A* (sip16) as a positive control (green bars) (Rapisarda et al., 2017) and determined BrdU incorporation and p21^CIP^ protein levels by IF (Figures 6G and S6B). From the primary screen, we selected the top 4 siRNAs that prevented cell-cycle arrest by BrdU and analyzed additional markers of senescence by IF—p16^INK4A^ and p-γH2AX (Figure S6C). The IFN signaling pathway has been recently described as regulating senescence (Yu et al., 2015), and within the siRNA validated from the screen, we found the IFN-inducible transmembrane protein 3 (IFITM3) (Figure 6G, red bars).

### IFITM3 within sEV Partially Mediates sEV-PS

**Figure 7:**
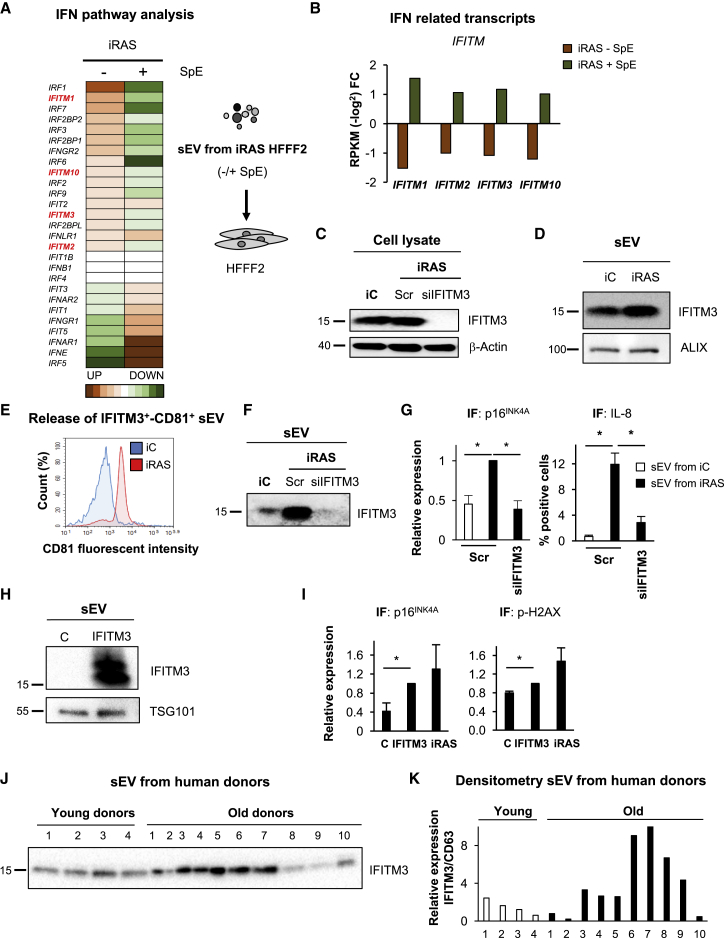
IFITM3 within sEVs Is Partially Responsible for Inducing Paracrine Senescence (A and B) HFFF2s incubated with sEVs derived from iRAS cells show an increase in transcripts related to the interferon (IFN) pathway (A)—in particular, *IFITM* (in red) and *IFIT* mRNAs, which are downregulated when treated with SpE. (B) *IFITM* transcripts are specifically shown. Data in (A) and (B) have been normalized to the control and represent the mean of 3 independent experiments (RPKM-log_2_ fold difference). (C and D) Immunoblotting analysis for IFITM3 in (C) cell lysates derived from iRAS HFFF2s transfected with Src or siIFITM3 and in (D) sEV (3 × 10^9^ particles) from iC and iRAS cells. β-Actin and ALIX are used as loading controls. (E) sEVs from iC and iRAS were captured onto IFITM3-coated beads and the presence of CD81-PE determined by FACS. (F) Immunoblotting showing the absence of IFITM3 in sEVs (3 × 10^9^ particles) derived from siIFITM3-treated cells. (G) IF staining for p16^INK4A^ and IL-8 in HFFF2s treated with the same number of sEV (1 × 10^7^ particles) derived from iC or iRAS transfected with or without siIFITM3. (H) Immunoblotting for IFITM3 present in sEVs derived from HFFF2 expressing an ectopic IFITM3 construct. (I) IF staining for p16^INK4A^ and p-γH2AX in HFFF2s treated with sEVs derived from cells expressing IFITM3. (J and K) Immunoblotting (J) and quantification (K) for IFITM3 and CD63 protein expression levels in sEVs derived from the plasma of young (∼33 years old) and old (∼80 years old) donors. Gels were loaded based on equal protein levels. (A–I) Data representative of >3 experiments. See also Figure S7.

Our previous data show that IFITM3 (1) is important for CM-mediated paracrine senescence (Figures 6G, S6B, and S6C), (2) has not been previously found to be a soluble factor (SN) in senescence (Figure 6E) (Acosta et al., 2013), and (3) is highly expressed in sEVs derived from cells undergoing OIS and DDIS (Figure S6A). To investigate whether the IFN pathway plays a role in sEV-PS, we analyzed the expression levels of RNA transcripts deregulated in HFFF2 treated with sEVs derived from OIS and DDIS cells (Figure 2). We found an increase in several *IFITM* and the related IFN-induced protein with tetratricopeptide repeats (*IFIT*) transcripts upon sEV incubation, but not when treated with N-SMase (Figures 7A, 7B, and S7A).

We next determined whether the increase in IFITM3 observed in the MS analysis was due to an increase in the endogenous expression levels of IFITM3 during senescence. To our surprise, the endogenous cellular levels of IFITM3 did not change during senescence in iRAS cells (Figures 7C and S7B), although we were able to detect an increase in IFITM3 in sEVs (Figure 7D). By capturing IFITM3^+^ sEVs onto beads and detecting CD81 by FACS, we confirmed that iRAS cells released more IFITM3^+^/CD81^+^ sEV particles than the iC cells (Figure 7E). To determine whether IFITM3^+^ sEVs belong to the same complex as CD63^+^ sEVs, we performed the OptiPrep density gradient and confirmed that they both float at the same density (Figure S7C), suggesting that sEVs containing IFITM3 are also positive for CD81 and CD63.

To determine the implication of IFITM3 in sEV-PS, we manipulated the levels of IFITM3 in donor cells, isolated sEV from these cells, and measured their ability to induce sEV-PS. We depleted IFITM3 from iRAS cells using siIFITM3, confirming a reduction in the expression levels of IFITM3 in the donor cells and their derived sEVs (Figures 7C, 7F, and S7B). The treatment of HFFF2 with an equal number of sEVs derived from iRAS + siIFITM3 prevented the upregulation of p16^INK4A^ and IL-8 mediated by sEVs from iRAS cells (Figure 7G). A similar response was observed using stable HFFF2 infected with a previously validated shRNA targeting *IFITM3* (Huang et al., 2011) (Figures S7D and S7E). However, there was no difference in the number of sEVs released from iRAS cells with or without both siIFITM3 and shIFITM3 (Figure S7F), confirming that the depletion of IFITM3 did not alter sEV particle secretion. Next, we generated HFFF2s expressing a lentiviral vector encoding an myc-tagged IFITM3 construct, in which we were able to confirm the ectopic expression of IFITM3 in both cell lysates and their respective sEVs (Figures 7H and S7G). In accordance with our previous data that the endogenous levels of IFITM3 do not change during senescence (Figure 7C), we did not observe any changes in the expression levels of a number of markers of senescence by immunoblot or qPCR (data not shown) upon the ectopic expression of IFITM3 in normal HFFF2s (Figure S7G) or changes in the release of a number of sEVs (Figure S7H). However, we could still observe a slight upregulation of p16^INK4A^ and an increase in p-γH2AX when we treated HFFF2 with sEVs isolated from IFITM3-expressing cells by IF, although these were not as prominent as the upregulation mediated by iRAS-derived sEVs (Figure 7I). Therefore, IFITM3 is not involved in regulating senescence, although changes in its expression levels within sEVs partially influence sEV-PS.

### IFITM3 Is Highly Expressed in sEVs Derived from Elderly Human Donors

To determine whether IFITM3 could be involved in aging, we isolated sEVs from human plasma derived from 4 young (∼33 years old) and 10 elderly (∼80 years old) donors and determined the expression levels of IFITM3 protein by immunoblotting. The expression levels of IFITM3 were increased in 6 of 10 elderly human donors, while there was very little change in the expression levels of the sEVs derived from young donors (Figures 7J, 7K, and S7I). Furthermore, the elderly human donors released more sEVs than did their younger counterparts (Figure S7J).

## Discussion

Intercellular communication is an important mechanism by which cells interact with one another. It can be mediated in the form of soluble factors or extracellular vesicles (Kuilman and Peeper, 2009, O’Loghlen, 2018, Tkach and Théry, 2016). However, although recent studies have highlighted the importance of EVs for cellular homeostasis in the context of senescence (Takahashi et al., 2017), a role for EVs has been neglected, in spite of some studies having previously found the “extracellular vesicle pathway” overrepresented by GO analysis of differentially expressed genes during senescence (Hoare et al., 2016, Lujambio et al., 2013).

Non-cell autonomous (paracrine) senescence via the SASP has been previously described as an important mechanism during senescence (Acosta et al., 2013, Dou et al., 2017, Hoare et al., 2016, Nelson et al., 2012), although these studies do not discern between the effect of soluble factors and EVs. Here, we provide evidence that both the SN and sEVs are responsible for mediating paracrine senescence (SN-PS and sEV-PS, respectively). In fact, a transcriptome analysis comparing SN-PS (Acosta et al., 2013) and sEV-PS shows a significant senescent signature. Furthermore, a broad MS analysis of the published protein composition of SN (Acosta et al., 2013) and the present article’s sEV proteomics shows little correlation between both fractions, suggesting that although the downstream signaling is similar, the triggers inducing senescence are diverse. However, a more comprehensive and sensitive methodology would be needed to confirm this. The use of a Cre *loxP* reporter system to determine EV internalization shows a positive correlation between sEV uptake and paracrine senescence activation.

Recently, many studies have found a cellular response that is characteristic of infectious agents during senescence in the absence of pathogens. For example, it has been shown that the SASP is regulated by the inflammasome via IL-1α signaling (Acosta et al., 2013) and by the cGAS-STING (cyclic guanosine monophosphate [GMP]-AMP synthase linked to the stimulator of IFN genes) pathway (Dou et al., 2017, Glück et al., 2017, Yang et al., 2017). Both pathways are induced during senescence in the absence of pathogens or double-stranded DNA. MS analysis of sEVs derived from OIS and DDIS show that IFITM3, which is implicated in the IFN signaling pathway, is accumulated in sEVs derived from both OIS and DDIS and is not detectable in the soluble fraction of the SASP (Acosta et al., 2013). IFITM3 endogenous expression levels are unchanged in donor cells during senescence but accumulate in sEVs derived from iRAS, which may suggest that IFITM3 could be selectively packaged into sEVs during OIS. We find that IFITM3 within sEVs is partially responsible for sEV-PS, as depletion of IFITM3 from sEVs using independent RNAi partially prevents sEV-PS. Conversely, the generation of a stable HFFF2 cell line ectopically expressing IFITM3 does not induce senescence in the donor cells, while sEVs derived from these cells are capable of inducing a DNA damage response and upregulation of p16^INK4A^. In fact, other investigators have found that IFN signaling can be mediated via EVs in neural stem cells (Cossetti et al., 2014, Li et al., 2013) and that DNA from senescent cells can be secreted via EVs (Takahashi et al., 2017). The relevance of individual components within EVs has also been described in different biological contexts such as cancer. EVs contain particular protein components, such as major histocompatibility complex (MHC) molecules, integrins, or the receptor kinase MET, that allow both immunosuppression and evasion of immune surveillance (O’Loghlen, 2018). However, that additional components of the senescent sEV (DNA, RNA, or lipids) could play a role in mediating sEV-PS cannot be dismissed.

Here, we show that PanINs, which are enriched in senescent cells, present a higher number of MVBs per cell than their *WT* counterparts. Furthermore, the increase in CD63 staining in regions enriched in senescent cells in human lung fibrotic tissues suggests that a positive correlation between CD63 endogenous expression and OIS occurs *in vivo*. It is interesting that elderly human donors release more sEVs and that the sEVs found in plasma show higher protein levels of IFITM3 in 60% of the elderly donors. Although it may be tempting to speculate that IFITM3 within sEVs could be involved in aging, a larger cohort of young and elderly patients would be needed.

We show here that sEVs are responsible for mediating paracrine senescence and speculate that they could be involved in inducing bystander senescence during therapy-induced senescence (Demaria et al., 2017) or aging (Acosta et al., 2013, Nelson et al., 2012). In fact, when compared to soluble factors, sEVs have different biophysical and biochemical properties as they have a longer lifespan than do soluble factors and they are more resistant to protease degradation (O’Loghlen, 2018). The idea that blocking sEV secretion could be a potential therapeutic approach to alleviate senescence “spreading” during chemotherapy-induced senescence or in aging tissues presents itself as a very attractive tool for the future.

## STAR★Methods

### Key Resources Table

**Table:** 

REAGENT or RESOURCE	SOURCE	IDENTIFIER
**Antibodies**		

p16^INK4A^	Abcam	Cat# ab108349, RRID:AB_10858268
β-Actin	Santa Cruz Biotech	Cat# sc-47778 HRP, RRID:AB_2714189
β-Actin	Abcam	Cat# ab8226, RRID:AB_306371
p21^CIP^	Abcam	Cat# ab109520, RRID:AB_10860537
BrdU	Invitrogen	Cat# A-21303, RRID:AB_221471
p53	Santa Cruz Biotech	Cat# sc-126, RRID:AB_628082
CD63	Abcam	Cat# ab68418, RRID:AB_10563972
CD63	BD PharMingen	Cat# 556019, RRID:AB_396297
CD81-PE	Life Technologies	Cat# A15781, RRID:AB_2534560
phospho-γH2AX	Merck Millipore	Cat# 05-636-I, RRID:AB_2755003
TSG101	Abcam	Cat# ab30871, RRID:AB_2208084
ALIX	Abcam	Cat# ab88743, RRID:AB_2042597
Ki67	Abcam	Cat# ab92742, RRID:AB_10562976
ANNEXIN V	Abcam	Cat# ab54775, RRID:AB_940268
IL-8	R&D Systems	Cat# MAB208, RRID:AB_2249110
IL-6	R&D Systems	Cat# AB-206-NA, RRID:AB_354281
IFTIM3	Abcam	Cat# ab109429, RRID:AB_10865792
COX IV	Abcam	Cat# ab14744, RRID:AB_301443
Vinculin	Sigma Aldrich	Cat# V4505, RRID:AB_477617
GAPDH	Abcam	Cat# ab9484, RRID:AB_307274
Calnexin	Abcam	Cat# ab22595, RRID:AB_2069006

**Biological Samples**		

Plasma from young and old individuals	This study	This study

**Chemicals, Peptides, and Recombinant Proteins**		

Spiroepoxide	Santa Cruz Biotech	sc-202721
GW4869	Sigma-Aldrich	D1692
TORIN-2	Cayman Chemical	14185
Doxycycline	Sigma-Aldrich	D9891
Palbociclib	APExBIO	A8316
4-hydroxytamoxifen	Sigma-Aldrich	T176
Aldehyde/sulfate latex beads	Thermo Fisher	A37304
Classical SA-β-Gal	Rapisarda et al., 2017	Rapisarda et al., 2017

**Critical Commercial Assays**		

IF SA-b-Gal	Sigma-Aldrich	F2756
Annexin V-FITC Apoptosis detection kit	ThermoFisher	A23204

**Deposited Data**		

RNA-seq	This paper	GSE131503
Proteomic data	This paper	PXD010379

**Experimental Models: Cell Lines**		

HFFF2	Culture Collections (Public Health England, UK)	86031405
BF	Rapisarda et al., 2017	Rapisarda et al., 2017
IMR-90	ATCC	CCL-186
HSC	Krizhanovsky et al., 2008	Krizhanovsky et al., 2008
MCF7	ATCC	Cat 30-2003
HEK293T	ATCC	CRL-3216

**Experimental Models: Organisms/Strains**		

*Pft1a*^*Cre*^*;IsI-Kras*^*G12D*^ mice	Helman et al., 2016	Helman et al., 2016

**Oligonucleotides**		

See Table S2 for primers	This paper	Table S2
siRNA: IFITM3	Dharmacon	M-014116-01
siRNA: MX1	Dharmacon	M-011735-00
siRNA: p16	QIAGEN	SI02623747
siRNA: p53	QIAGEN	SI02664403

**Recombinant DNA**		

pLenti6-mCherry-CD63	Alissa M. Weaver	Alissa M. Weaver
pcDNA3.1-CMV-CFP;UBC-Cre25nt	Addgene	65727
pLV-CMV-LoxP-DsRed-LoxP-eGFP	Addgene	65726
pKLO puro-shp16	O’Loghlen et al., 2015	O’Loghlen et al., 2015
pRS hygro-shp53	Rapisarda et al., 2017	Rapisarda et al., 2017
pRS puro – shIFITM3 (human)	Huang et al., 2011	Huang et al., 2011
pLenti CMV Puro – c-myc-IFITM3	Jacob S. Yount	Jacob S. Yount
ER:RAS^G12V^	Rapisarda et al., 2017	Rapisarda et al., 2017

**Software and Algorithms**		

STRING: functional protein association networks	STRING	https://string-db.org
DAVID Functional Annotation Bioinformatics Microarray Analysis	DAVID	https://david.ncifcrf.gov
PANTHER - Gene List Analysis	PANTHER	http://www.pantherdb.org
GSEA	GSEA	https://software.broadinstitute.org/gsea/index.jsp

**Other**		

Transwell Chambers	Thermo Fisher	141002
NTA Calibration Beads (100 nm)	Polyscience	24041
10k protein concentration columns	EMD Millipore	10088753
ELISA IL-8	Mab Tag GmbH	h-IL8-EIA-5
ELISA IL-6	Mab Tag GmbH	h-IL6-EIA-1
ELISA TGF-β	R&D systems	DB100B
β-Galactosidase Staining kit	Cell Signaling	#9860

### Lead Contact and Materials Availability

Further information and requests for resources and reagents should be directed to and will be fulfilled by the Lead Contact, Ana O’Loghlen (a.ologhlen@qmul.ac.uk).

### Experimental Model and Subject Details

#### Cell culture

HFFF2 human foreskin primary fibroblasts (male) were obtained from the Culture Collections (Public Health England, UK). IMR-90 (female), MCF7 (female) and HEK293T (female) were bought from ATCC. Breast fibroblasts (BF) were isolated from a female breast mastectomy and have been described elsewhere (Rapisarda et al., 2017). All cells were grown in high glucose Dulbecco’s modified Eagle’s medium with 10% fetal bovine serum and 1% antibiotic-antimycotic solution. Mouse hepatic stellate cells were a kind gift from Scott Lowe and were grown in 1 μg/ml of Doxycycline.

#### Mice samples

6 week-old *Ptf1a*^*Cre*^*;lsl-Kras*^*G12D*^ mice were injected subcutaneously with two doses, two days apart, of 400 μg tamoxifen per 20 g mice (Tamoxifen (Sigma) stock 20 mg/ml in corn oil) to obtain acinar cell-specific activation of *KrasG12D*. Mice were euthanized 5 months following treatment. All mice experiments were done with approval from the Hebrew University Animal Care and Use Committee.

#### Human EV plasma samples

Young donors were all male with an age range between 29-36 years old, while old donors were a mix between male and female and age range between 70-92. None of the donors presented underlying diseases. The study was approved by the London - City & East Committee (10/H0704/73) and all donors gave informed written consent to participate.

#### Human lung samples

Human tissues were received from the Papworth Hospital Research Tissue Bank (REC 08/H0304/56+5).

### Method Details

#### Soluble fraction, MV and sEV isolation

All cells were maintained in sEV-depleted FBS. FBS was depleted of sEV by overnight (ON) ultracentrifugation at 100,000 g at 4°C (Sorvall 100SE Ultracentrifuge). The supernatant was removed and stored in 50 mL falcons at −20°C until required. CM was collected after 72h incubation with cells, unless specified otherwise.

To isolate the different EV fractions, the protocol of differential ultracentrifugation (Théry et al., 2006, Théry et al., 2018) was modified and adapted. Whole CM was centrifuged at low speed (2,000 g for 20min) to eliminate dead cells and cellular debris prior to use. MV were collected after the 10,000 g centrifugation step for 1h, washed with PBS and spun down again at 10,000 g for 1h. The supernatant was then filtered through a 0.22μm filter prior to the 100,000 g centrifugation step and also after in some cases (for the Cre-LoxP experiments). The supernatant was collected after a 1h and 20min 100,000 g centrifugation step and concentrated using a 10K column (Amicon Ultra-0.5 Filter) at 14,000 g for 10min obtaining a concentration factor 10X. The final 100,000 g pellet was washed once in 15ml of PBS and resuspended in 100 μL of 10% FBS-depleted media for the functional cell culture experiments. For the MCF7 functional experiments the pellet was resuspended in 0.5% FBS-depleted media. Alternatively, for Western Blot analysis, sEV pellet was re-suspended in protein lysis buffer. A Sorvall 100SE Ultra Centrifuge, with a Beckmann Fixed Angle T865 rotor was used for all sEV isolations. The k-factor of the rotor is 2,08. We have submitted all relevant data of our experiments to the EV-TRACK knowledgebase (EV-TRACK ID: EV190024).

#### Density gradient sEV isolation

sEV isolated by serial ultracentrifugation were re-suspended in 1.5 mL of suspension buffer (0.25M sucrose, 10mM Tris pH 8.0 and 1mM EDTA (pH 7.4) (Sigma-Aldrich, USA). Next, sEV were mixed 1:1 with 60% stock solution of iodixanol/Optiprep (Sigma-Aldrich, USA). Then, 1.4ml 40% iodixanol, 1.3ml 20% iodixanol and 1.2ml 10% iodixanol were successively layered on top of the sEV suspension and tubes were centrifuged at 100,000 g ON, stopping without break. After centrifugation, ten fractions of 700μl were collected from the top of the tube. Fractions were washed with 15ml PBS at 100,000 g for 1h 20 min. The fractions were re-suspended in 50 μL of lysis buffer.

#### Size exclusion sEV isolation

CM was centrifuged at 350 g for 15min at 4°C to pellet cells. The supernatant was centrifuged at 2,000 g for 20min at 4°C and filtered with a 0.22μm filter. Size exclusion chromatography (SEC) using qEV columns (Izon Science, USA) was used to the isolate sEV. Twelve eluted fractions were collected in sequential fractions of 1ml according to the manufacturer’s instructions. The particle and protein concentration of each fraction was then measured by NTA and MicroBCA. The fractions enriched in particles and lacking protein contaminants were pooled, centrifuged at 100,000 g for 1h 20 min and used for functional assays.

#### Treatment of cells with CM or isolated sEV

HFFF2 fibroblasts were plated in a 100mm dish at 1x10^6^ cells. After 24h, iC and iRAS cells were treated with 200nM 4OHT for 48h in 10% media, washed, incubated with 0.5% media and allowed to produce new CM for 72h. The sEV obtained from these cells were used to treat 10-12 wells of a 96-well plate.

Young HFFF2 were treated with the CM from several experiments previously centrifuged at low speed to discard dead cells and supplemented with FBS to reach 10% in the final volume. HFFF2 treated with isolated sEV were also supplemented with media containing 10% FBS, while in the experiments performed with MCF7, the cells were incubated with isolated exosomes resuspended in 0.5% FBS media.

#### CM with siRNA and inhibitors functional experiments

24h after plating HFFF2 in 96-well plates, senescence was induced by adding 200nM 4OHT. 48h after the cells were washed and supplemented with 0.5% FBS and the indicated inhibitors for 2-3 days, after which plates were washed again to remove the inhibitors from the media and supplemented with fresh media (0.5% FBS) for further 72h. For the experiments with the siRNA, reverse transfection with 50nM siRNA was performed and the media replenished (10% FBS) with 4OHT after 2 days and left for an additional 48h. After, the cells were washed to remove all siRNA from the media and incubated with 0.5% FBS fresh media for an additional 72h.

#### Nanoparticle tracking analysis (NTA)

Prior to the NTA analysis, the NanoSight LM10 equipped with a 405nm laser (Malvern Instruments) was calibrated using Silica Microspheres beads (Polyscience). Samples to be measured were then diluted in PBS in order to obtain a particle number between 10^8^-10^9^ particles. At least three repeated-measurements of 60 s were taken per each individual sample and the mean value was used to determine particle number. Static mode (without flow) was used for each analysis. The movement of each particle in the field of view was measured to generate the average displacement of each particle per unit time which was calculated using the NTA 3.0 software.

#### Affinity-based capture of exosomes on beads

For exosome characterization by flow cytometry, aldehyde/sulfate latex beads (Thermo Fisher) were coated with anti-CD63 or anti-IFITM3 antibody and incubated with the different CM overnight at 4°C in a rotation wheel. After extensive washing, anti-CD81-PE conjugated antibody was added for further 40min at RT, washed with PBS and acquired using NovoCyte Flow Cytometer (Acea, Biosciences) with a 488nm laser. Gates were set using the NovoExpress Software (Acea, Bio) to analyze single bead fluorescence. Isotype-matching coated beads were used as a negative control in all experiments.

#### FACS sorting of GFP^+^ and DsRed+ MCF7 cells

Reporter MCF7 incubated with sEV isolated from DMSO and Palbo treated Cre^+^ MCF7, were washed twice with PBS, analyzed and sorted into two populations (GFP^+^ and DsRed^+^) using FACS Aria Ill Cell Sorter (BD Biosciences). Cell debris was discriminated by the cell forward scatter (FSC) and side scatter (SSC) properties. FACS data sorter was generated using BD FACDIVA SoftwareTM v.8.0.1 (BD Biosciences).

#### Transmission Electron Microscopy

##### Mouse pancreatic tissue

Mice were perfused with PBS followed by using fixative solution (2% PFA / 2.5% glutaraldehyde / 0.1M cacodylate buffer). Small tissue sections were dissected and placed under rotation in fixative solution for 2h at RT, followed by ON rotation at 4°C. After 2 days, the tissue was transferred to 0.1M cacodylate buffer, pH 7.4. After fixation, samples were rinsed several times with 0.1 M cacodylate buffer and post-fixed in 1% (v/v) osmium tetroxide in 0.1 M cacodylate buffer (pH 7.4) for 1.5h at 4°C. Samples were then en-bloc stained with 1% (w/v) aqueous uranyl acetate for 1h at RT, thoroughly washed and dehydrated through a graded ethanol series before infiltration with epoxy resin (TAAB). Finally, tissue samples were embedded on flat molds and polymerized at 70°C for 24h. Ultrathin sections (70-90nm) were cut using a Leica UC7 ultramicrotome mounted on 150 mesh copper grids and contrasted using Uranyless (TAAB) and 3% Reynolds Lead citrate (TAAB). Sections were examined at 120kV on a JEOL JEM-1400Plus TEM fitted with a Ruby digital camera (2k x 2k).

##### Isolated sEV

2.5 μL of sEV resupended in PBS were placed on Formvar-coated grids and allowed to settle for 3-5min, without being allowed to dry. sEV were then fixed with 2% glutaraldehyde for 5min and washed three times with distilled de-ionised water. After washing, the grids were stained for 20min with 3% uranyl acetate: 2% methyl cellulose (1:9). Imaging of sEV was carried out using a JEOL JEM-1400Plus, operated at 120kV, fitted with a Ruby camera (2k x 2k).

#### β-Galactosidase staining

Cells were washed with PBS and fixed with 0.05% (w/v) glutaraldehyde (in PBS) for 15mins at RT. Cells were washed a second time with PBS and incubated with 5-bromo-4-chloro-3-indolyl-beta-D-galacto-pyranoside (X-gal) solution for 1h at 37°C. Cells were imaged after 12-24h using a light microscope (Nikon) at 20X magnification and single representative images of each well were taken. IF β-Galactosidase was performed according to the manufacturer’s instructions using the following commercial kit (Sigma-Aldrich, #F2756). Briefly, 33 μM of the β-gal substrate C12FDG (Fluorescein di-B-D-galactopyranose) (F2756 Sigma-Aldrich) was added to the cells for 8h at 37°C, After, the cells were washed with PBS and fixed with 4% PFA.

#### IF staining

Cells grown in 96-well plates were washed with PBS and fixed in 4% paraformaldehyde for 15min at RT. Cells were then washed in PBS twice before been permeabilized and blocked for 40min with 0.2% Triton X-100 together with 1%BSA and 0.2% gelatin fish (Sigma). For IF staining, cells were incubated ON with the primary antibody and in the case of BrdU cell were treated with 0.5U/μl DNaseI and 3mM MgCl_2_. Cells were then washed in PBS and incubated 1h with secondary antibody, DAPI and Cell Mask Deep Red (Invitrogen). For confocal images, cells were mounted onto slides using Vectashield Mounting Medium (Vector Laboratories). Signals were visualized using a Zeiss LSM 880 (Zeiss, Berlin, Germany) provided with Airyscan for super-resolution acquisition.

#### RNA extraction, cDNA synthesis and qPCR

Cells were washed with PBS and lysed directly into the culture dish using TRIzol Reagent (Thermo Fisher). cDNA synthesis was performed using the High-Capacity cDNA Reverse Transcription Kit (Thermo Fisher). qPCR reactions were performed using SYBR Green PCR Master Mix (Applied Biosystems,) on a 7500 Fast System RealTime PCR cycler (Applied Biosystems). Primer sequences are listed in Table S2.

#### ELISA

The SN sample was diluted 1:2 prior to the analysis and sEV - isolated by ultracentrifugation - were suspended in 100μl of lysis buffer to determinate the concentration of TGF-β1, IL-6 and IL-8. Regarding TGF-β1, it was first activated in the samples. All samples were incubated with 1N HCl for 10min at RT. Then, the acidified samples were neutralized with 1.2 N NaOH/0.5 M HEPES. TGF-β1 was detected using the quantitative sandwich human TGF-β1 immunoassay (DB100B, R&D systems) and human IL-6 and IL-8 immunoassays (h-IL6-EIA-1 and h-IL8-EIA-5 respectively, Mab Tag GmbH). Samples were measured at 540nm using Synergy HT Multi-Mode Microplate Reader.

#### Stable gene expression

Stable retroviral and lentiviral expression was performed as in previous studies (Acosta et al., 2008, Rapisarda et al., 2017).

#### Protein analysis by immunoblotting

sEV and cultured cells were lysed using the following lysis buffer [(Tris-HCl 20 mM pH 7.6; DTT 1 mM; EDTA 1 mM; PMSF 1 mM; benzamidine 1 mM; sodium molybdate 2 mM; β- sodium glycerophosphate 2 mM; sodium orthovanadate 0.2 mM; KCl 120 mM; 1 μg/ml (each) leupeptin, pepstatin A and antipain; Nonidet^TM^ P-40 0.5% (v/v); Triton X-100 0,1% (v/v)], and quantified using a Micro BCA Protein Assay Kit (Thermo Fisher Scientific) or cell lysates were prepared with Lysis Buffer 6 (R&D Systems), supplemented with protease inhibitor cocktail (Thermo Fisher Scientific), and protein content was determined using the Advanced Protein Assay Reagent (Sigma-Aldrich). Lysates were diluted with 4X Laemmli Sample Buffer (Bio-Rad, UK) and equal quantities of total protein were separated in SDS-PAGE gels, transferred to a PVDF 0.45 μm pore size membrane (Millipore, UK) and probed with different antibodies. Protein bands were detected using a SuperSignal West Pico PLUS Chemiluminescent Substrate (Thermo Fisher Scientific) and the ChemiDoc XRS+ System (Bio-Rad). Sequential detection of different proteins was performed following HRP inactivation with 30% H_2_O_2_ (Sigma-Aldrich) for 30 minutes at 37°C up to five times, or without inactivation where appropriate.

#### Immunohistochemistry of human lung samples

SA-β-Gal staining was performed in whole tissue, using the Senescence β-Galactosidase Staining kit (Cell Signaling #9860), following the manufacturer instructions. Briefly, whole tissue was fixed at RT for 45min with a 2% formaldehyde and 0.2% glutaraldehyde, washed and incubated ON at 37°C with the staining solution containing X-gal in N-N-dimethylformamide (pH 6.0). Tissues were subsequently dehydrated and embedded in paraffin and sectioned. For the immunohistochemistry, 5μm paraffin sections were deparaffinized and re-hydrated, and slides were incubated with anti-CD63 (Cell Signaling #55051S). The immunohistological reaction was developed using 3,3-diaminobenzidine tetrahydrochloride (DAB), and nuclei counterstained with hematoxylin. Positive signal for SA-β-Gal and CD63 was quantified with ImageJ.

#### RNA sequencing

For RNA-seq, total RNA was extracted using TRIzol Reagent. First strand cDNA synthesis was performed with SuperScript III First-Strand Synthesis System. After purification using SPRI beads, the double stranded cDNA was ligated to in-house designed adapters (based on TruSeq Indexed adapters (Illumina)) using NEBNext Ultra II (NEB) followed by 15 cycles of amplification and library purification. Sequencing was performed on an Illumina NextSeq500, High Output run with 75bp paired-end at the Genomics Centre (QMUL).

#### MS proteomics

LC-MS/MS was done by coupling a nanoLC-Ultra 1D+ system (Eksigent) to an Impact mass spectrometer (Bruker) via a Captivespray source (Bruker) supplemented with a nanoBooster operated at 0.2 bar/min with isopropanol as dopant. Peptides were loaded into a trap column (NS-MP-10 BioSphere C18 5 μm, 20 mm length, NanoSeparations) for 10min at a flow rate of 2.5 μl/min in 0.1% FA. Then peptides were transferred to an analytical column (ReproSil Pur C18-AQ 2.4 μm, 500 mm length and 0.075 mm ID, Dr. Maisch) and separated using a 100 min effective curved gradient (buffer A: 4% ACN, 0.1% FA; buffer B: 100% ACN, 0.1% FA) at a flow rate of 250 nL/min. The gradient used was: 0-2min 2% B, 2-102 min 33% B, 102-112 min 98% B, 112-120 min 2% B. The peptides were electrosprayed (1.35 kV) into the mass spectrometer with a heated capillary temperature of 180°C. The mass spectrometer was operated in a data-dependent mode (130-1600 m/z), with an automatic switch between MS and MS/MS scans using a top 20 method (threshold signal ≥ 500 counts, z ≥ 2 and m/z ≥ 350). An active exclusion of 30 s was used. The precursor intensities were re-evaluated in the MS scan (n) regarding their values in the previous MS scan (n-1). Any m/z intensity exceeding 5 times the measured value in the preceding MS scan was reconsidered for MS/MS. Peptides were isolated using a 2 Th window and fragmented using collision induced dissociation (CID) with a collision energy of 23-56 eV as function of the m/z value.

#### Human donors sEV isolation

10ml of blood was collected in EDTA vacutainer tubes (Nucare, VS367525). Plasma was obtained by centrifuging the tubes for 5min at RT at 1500 rpm. sEV were isolated from 2ml of plasma and centrifuged at 4,000 g for 10min at 4°C. Platelet-free plasma was filtered (0.22μm) and ultracentrifugated at 100,000 g for 1h 20min. After, the pellet was washed two times with PBS at 100,000 g for 1h 20min. None of the donors presented any underling disease.

### Quantification and Statistical Analysis

#### qPCR gene expression

Ct values were generated using the 7500 software version 2.0.6 (Applied Biosystems). Relative gene expression was calculated using the ΔΔCt method and normalized to a housekeeping gene, RPS14. The relative mRNA expression level changes were expressed as a fold change relative to the control or the senescent sample.

#### IF analysis

Immunofluorescence images were acquired using IN Cell 2200 automated microscope (GE) and the IN Cell 2200 Developer software version 1.8 (GE) as previously (Acosta et al., 2008, Rapisarda et al., 2017). Regarding the confocal images, rotating 3D reconstruction of confocal Z stack images were obtained using Zeiss Zen 2011 software.

#### MS proteomics analysis

Raw files were processed with MaxQuant (v 1.5.3.30) using the standard settings against a human protein database (UniProtKB/Swiss-Prot, August 2016, 20,195 sequences) supplemented with contaminants. Label-free quantification was done with match between runs (match window of 0.7 min and alignment window of 20 min). Carbamidomethylation of cysteines was set as a fixed modification whereas oxidation of methionines and protein N-term acetylation as variable modifications. Minimal peptide length was set to 7 amino acids and a maximum of two tryptic missed-cleavages were allowed. Results were filtered at 0.01 FDR (peptide and protein level). Further statistical analysis was performed using Perseus (v1.5.5.2). A minimum of three LFQ valid values per group was required for quantification. Missing values were imputed from the observed normal distribution of intensities. Then, a two-sample Student’s T-Test with a permutation-based FDR was performed. Only proteins with a q-value < 0.10 and log_2_ ratio > 2 or < −2 were considered as regulated.

#### RNA sequencing

Genomic mapping was performed by QMUL Genome Centre using Fastq files aligned to HG 19 using STAR aligner implemented in BaseSpace (RNA-Seq Alignment pipeline v 1.1.0, Illumina). BAM file outputs from STAR were annotated using Partek Genomic Suite (v6.6) and the RefSeq data base (RefSeq 21). Differential analysis was performed with Partek Genomic Suite (v6.6) running ANOVA.

#### Statistical analyses

Statistical analysis was performed using Student’s t test except were specified. P values represent the following: ^∗^ p < 0.05; ^∗∗^ p < 0.01; ^∗∗∗^ p < 0.001

### Data and Code Availability

#### Gene Ontology Analysis

Gene Ontology Analysis was performed using STRING: functional protein association networks (https://string-db.org/) for the proteomic dataset. For the RNA seq datasets a combination of DAVID Functional Annotation Bioinformatics Microarray Analysis (https://david.ncifcrf.gov/) and PANTHER - Gene List Analysis (http://www.pantherdb.org/) was used. Gene Set Enrichment Analysis (GSEA) was performed using the GSEA software (Broad Institute of the Massachusetts Institute of Technology (MIT) and Harvard (https://www.broadinstitute.org/gsea)).

#### Repository Information and Accession Numbers

The accession number for the sequencing data reported in this paper is GEO: GSE131503.

The mass spectrometry proteomics data have been deposited to the ProteomeXchange Consortium via the PRIDE partner repository with the dataset identifier PXD: 010379.

## Supplemental Information

Document S1. Figures S1–S7 and Tables S1 and S2

Document S2. Article plus Supplemental Information
