# Supplementary material for: Small Extracellular Vesicles Are Key Regulators of Non-cell Autonomous Intercellular Communication in Senescence via the Interferon Protein IFITM3
Source: Cell Rep. 2019 Jun 25;27(13):3956–3971.e6. doi: 10.1016/j.celrep.2019.05.095 (PMC6613042; doi:10.1016/j.celrep.2019.05.095)
Supplement: Document S1. Figures S1–S7 and Tables S1 and S2 [file mmc1.pdf]

Cell Reports, Volume *27*

Supplemental Information

Small Extracellular Vesicles Are Key Regulators of Non-cell Autonomous Intercellular Communication in Senescence via the Interferon Protein IFITM3

Michela Borghesan, Juan Fafián-Labora, Olga Eleftheriadou, Paula Carpintero-Fernández, Marta Paez-Ribes, Gema Vizcay-Barrena, Avital Swisa, Dror Kolodkin-Gal, Pilar Ximénez-Embún, Robert Lowe, Belen Martín-Martín, Hector Peinado, Javier Muñoz, Roland A. Fleck, Yuval Dor, Ittai Ben-Porath, Anna Vossenkamper, Daniel Muñoz-Espin, and Ana O'Loghlen

Borghesan *et al.* **Figure S1**

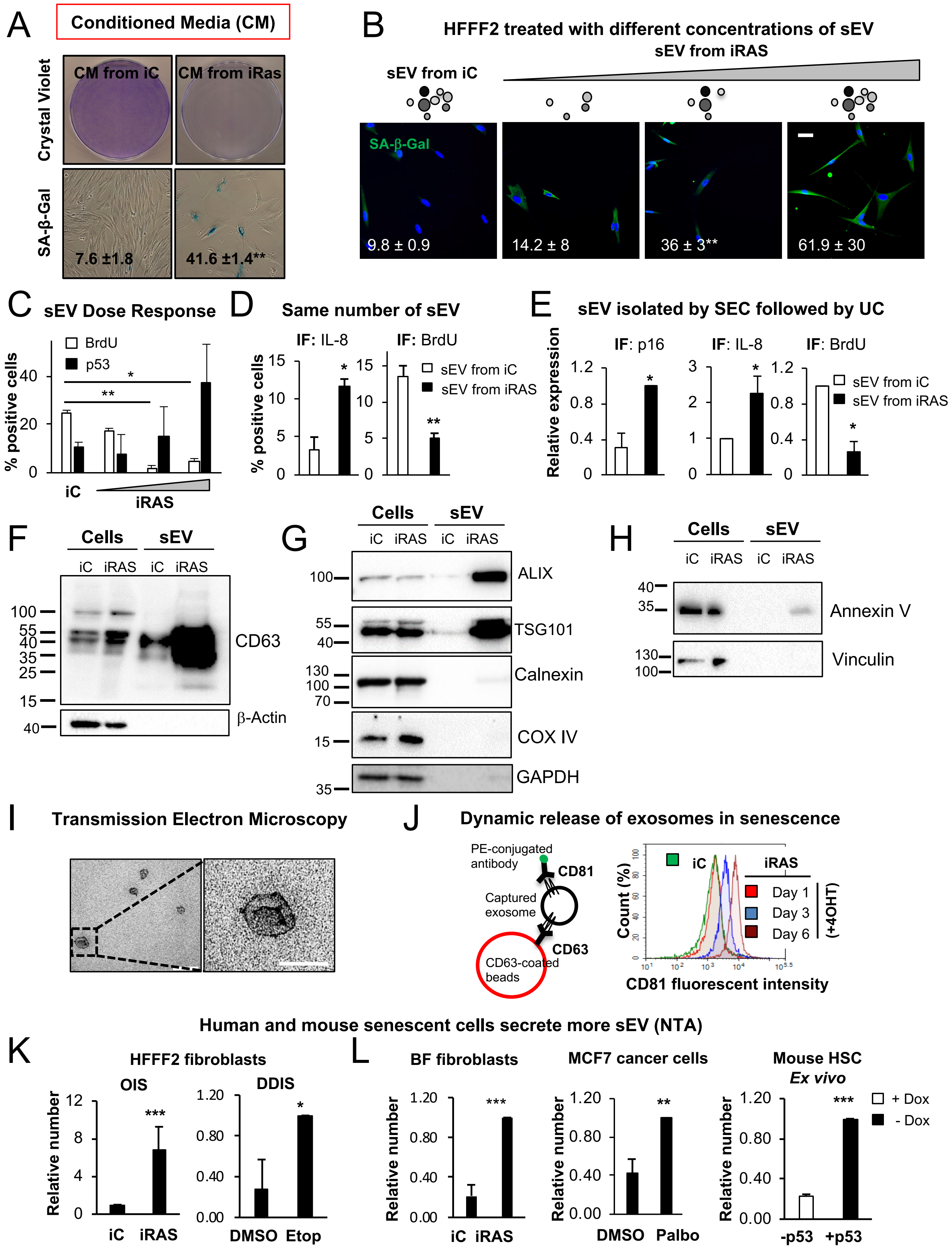

Figure S1. Confirmation of sEV-mediated paracrine senescence and exosome-like particle characterization within sEV. Related to Figure 1

(**A**) HFFF2 fibroblasts were treated with whole conditioned media (CM) derived from iC or iRAS HFFF2 for 2 weeks to confirm the induction of paracrine senescence. The cell density and proliferation are shown by crystal violet staining (upper panel) and the induction of senescence by staining for the activity of senescence-associated β-galactosidase (SA-β-gal) (lower panel). A representative experiment is shown. (**B,C**) HFFF2 fibroblasts were treated with different concentrations of sEV purified from iRAS cells (diluted 1:5, 1:2.5 and not diluted). Non-diluted sEV were used in the iC. (**B**) Staining for SA-β-Gal activity by IF. Numbers indicate the percentage of cells staining positive for SA-β-Gal. Scale bar, 40μm. (**C**) Graph representing the percentage of HFFF2 cells staining positive for BrdU or p53 treated with different concentrations of sEV from iRAS HFFF2. Graph is showing the mean ± SD of 2 independent experiments. (**D**) HFFF2 fibroblasts were treated with the same number of sEV ($1x10^7$ particles) and the percentage of cells incorporating BrdU and expressing IL-8 was determined by IF. Graphs represent the mean ± SD of 2-4 independent experiments. (**E**) sEV were isolated by performing size exclusion chromatography (SEC) followed by serial ultracentrifugation and sEV-PS was determined. Mean ± SD of 3 experiments. (**F-H**) Immunoblotting staining for different exosome-related proteins (CD63, ALIX, TSG101, AnnexinV) and cytoplasm contaminants (Calnexin and COX IV). Samples were loaded by protein content. β-Actin, GAPDH and Vinculin were used as loading controls. (**I**) Negative staining Transmission electron microscopy (TEM). **Left image:** overview of the shape and size of isolated sEV. Scale bar, 200nm. **Right image**: zoomed image showing a single sEV. Scale bar, 100nm. (**J**) **Left panel**: schematic representation of the exosomes-beads capture protocol used. Exosomes were captured onto latex-beads coupled with a CD63 antibody and detected with a CD81-PE antibody. **Right panel**: $CD63^+/CD81^+$ exosomes were then quantified by FACS from the CM of iC or iRAS cells treated with 200nM 4OHT for 1, 3 and 6 days. A representative experiment is shown. (**K**) The number of sEV released by HFFF2 undergoing OIS and DDIS was determined using NTA. OIS and DDIS was established by treating iRAS with 200nM 4OHT (OIS) or HFFF2 fibroblasts with 50μM Etoposide (DDIS) for 2 days followed by 5 days with fresh media. (**L**) NTA particle analysis for sEV isolated from breast fibroblasts (BF) infected with either a control vector (iC) or an inducible vector encoding ER:H-RAS^{G12V} (iRAS) –**left graph**; MCF7 breast cancer cells undergoing senescence by treatment with 500nM Palbo for 10 days – middle graph and; hepatic stellate cells (mHSC) derived from a mouse expressing a Doxycycline (Dox) inducible shp53 with or without Dox for 5 days – **right graph**. Data represents the mean ± SD of 2-5 independent experiments.

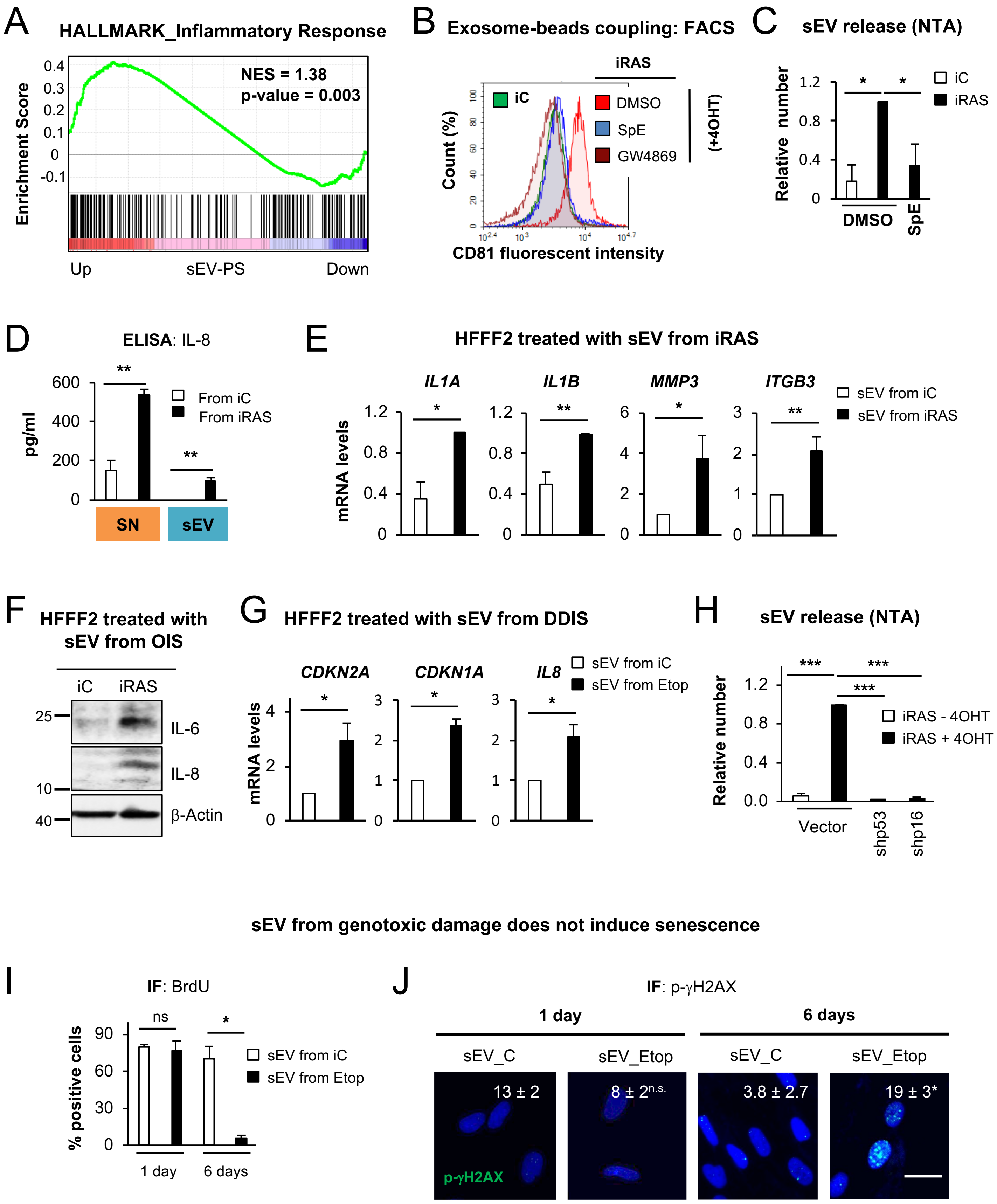
A
HALLMARK_Inflammatory Response
NES = 1.38
p-value = 0.003
Enrichment Score
Up
sEV-PS
Down
B
Exosome-beads coupling: FACS
iRAS
iC
DMSO
SpE
GW4869
(+4OHT)
Count (%)
CD81 fluorescent intensity
C
sEV release (NTA)
iC
iRAS
Relative number
DMSO
SpE
D
ELISA: IL-8
pg/ml
From iC
From iRAS
SN
sEV
E
HFFF2 treated with sEV from iRAS
IL1A
IL1B
MMP3
ITGB3
sEV from iC
sEV from iRAS
mRNA levels
F
HFFF2 treated with sEV from OIS
iC
iRAS
IL-6
IL-8
β-Actin
G
HFFF2 treated with sEV from DDIS
CDKN2A
CDKN1A
IL8
sEV from iC
sEV from Etop
mRNA levels
H
sEV release (NTA)
iRAS - 4OHT
iRAS + 4OHT
Relative number
Vector
shp53
shp16
sEV from genotoxic damage does not induce senescence
I
IF: BrdU
% positive cells
sEV from iC
sEV from Etop
1 day
6 days
J
IF: p-γH2AX
1 day
6 days
sEV_C
sEV_Etop
13 ± 2
8 ± 2n.s.
3.8 ± 2.7
19 ± 3*
p-γH2AX

Figure S2. sEV isolated from senescent cells induce paracrine senescence. Related to Figure 2
(**A**) Gene Set Enrichment Analysis (GSEA) for genes involved in an "Inflammatory Response" pathway in HFFF2 treated with sEV derived from Etop-treated fibroblasts. (**B**) $CD63^{+}/CD81^{+}$ exosomes were then quantified by exosome-beads capture by FACS in iC or iRAS cells treated with 200nM 4OHT for 6 days followed by DMSO or 10μM GW4869 or 5μM SpE. A representative experiment is shown. (**C**) NTA analysis of iRAS cells treated with 5μM SpE. (**D**) ELISA for IL-8 concentration in the SN or sEV lysed fraction derived from iC and iRAS HFFF2. (**E-G**) HFFF2 treated with sEV derived from OIS and DDIS cells upregulate different markers of senescence as measured by (**E,G**) qPCR or (**F**) immunoblotting. (**H**) NTA analysis of sEV released in iRAS cells treated with or without 4OHT expressing a vector encoding shp53 or shp16. One-way ANOVA analysis was performed. (**I,J**) sEV from cells treated for 1 or 6 days with Etop were isolated and used to treat normal HFFF2. (**I**) Proliferation and (**J**) DNA-damage was determined by IF in HFFF2 treated with sEV collected at the different time points. Scale bar, 40μm. All data represent the mean ± SD of 2-4 experiments.

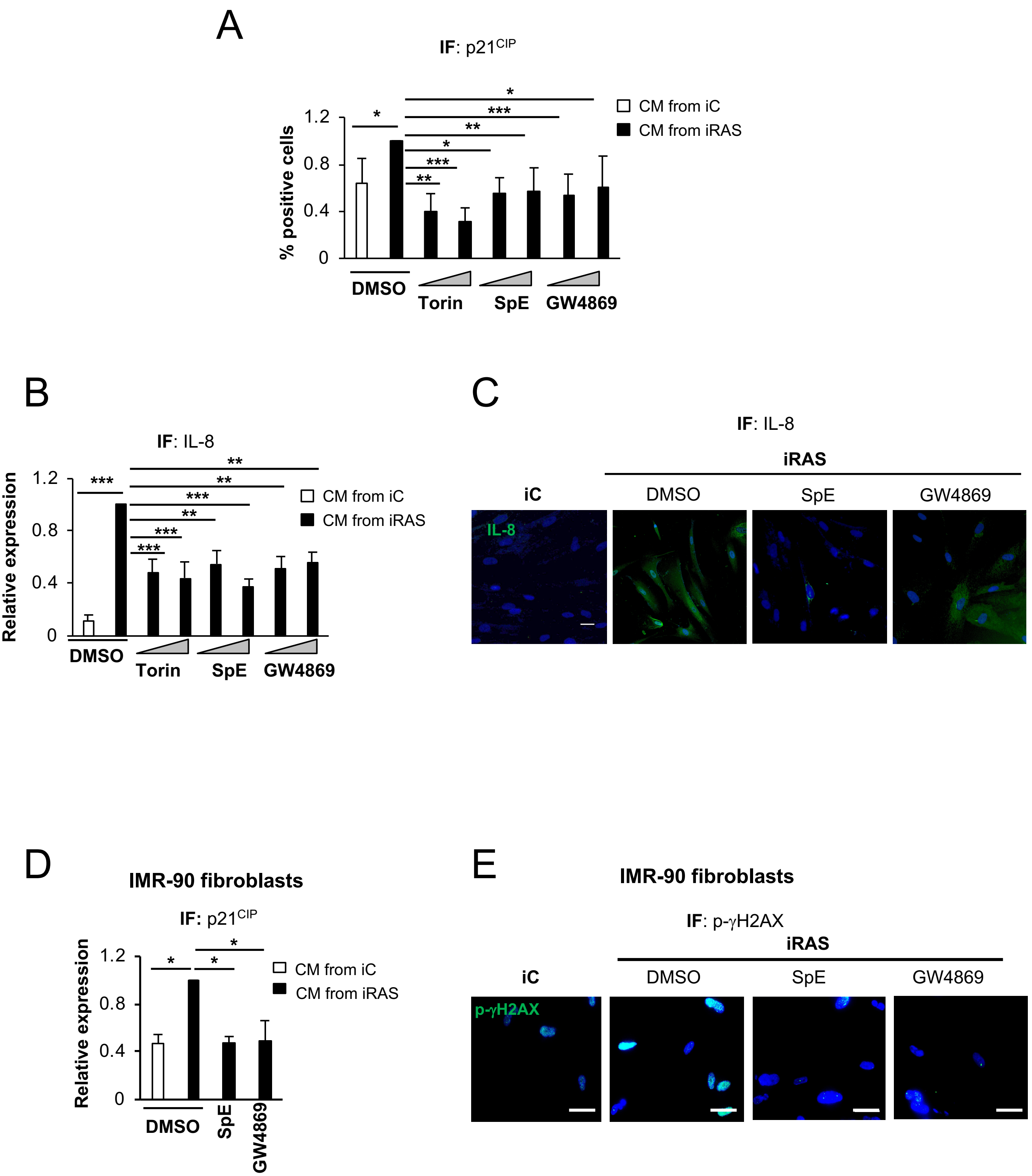
A
IF: p21CIP
% positive cells
1.2
0.8
0.4
0
*
***
**
*
***
**
*
CM from iC
CM from iRAS
DMSO
Torin
SpE
GW4869
B
IF: IL-8
Relative expression
1.2
0.8
0.4
0
***
**
**
***
**
***
***
CM from iC
CM from iRAS
DMSO
Torin
SpE
GW4869
C
IF: IL-8
iRAS
iC
DMSO
SpE
GW4869
IL-8
D
IMR-90 fibroblasts
IF: p21CIP
Relative expression
1.2
0.8
0.4
0
*
*
*
CM from iC
CM from iRAS
DMSO
SpE
GW4869
E
IMR-90 fibroblasts
IF: p-γH2AX
iRAS
iC
DMSO
SpE
GW4869
p-γH2AX

Figure S3. Evaluation of additional markers of senescence upon the inhibition of N-SMase. Related to Figure 3

(**A-C**) HFFF2 were incubated with CM from iRAS cells with or without SpE or GW4869 and the levels of (**A**) $p21^{CIP}$ and (**B,C**) IL-8 were determined by IF. Scale bar, 30μm. (**D,E**) Similar experiments were performed in a different strain of human primary fibroblasts, IMR-90, where (**D**) $p21^{CIP}$ protein expression levels and (**E**) p-γH2AX were determined by IF. Scale bar, 50μm. (**A-D**) One-way ANOVA was performed for the statistical analyses.

A

IHC: CD63/SA-β–Gal

Fibrotic sarcoidosis lung

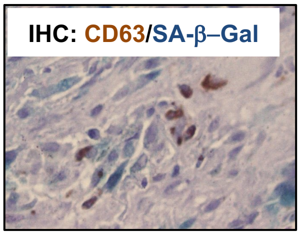

Hypersensitivity pneumonitis

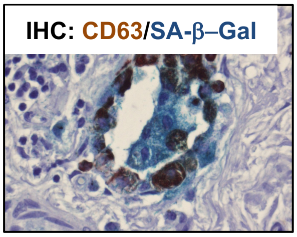

B

PanIN present different features of senescent cells

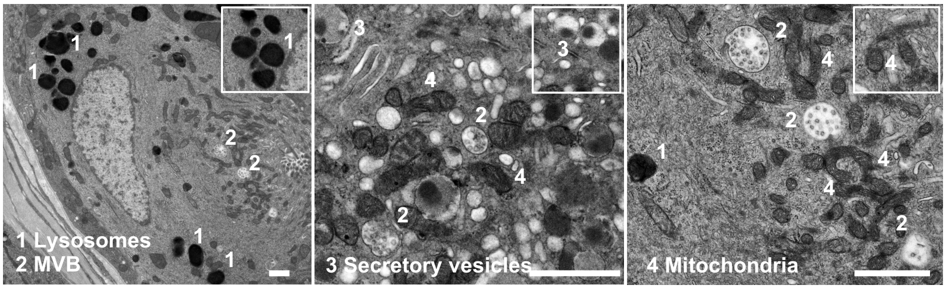

Figure S4. Increase in different features of senescence in human and mouse samples *in vivo*. Related to Figure 4

(**A**) Immunohistochemistry for CD63 show a positive correlation between cells staining positive for SA–β-Gal and CD63 in human tissue sections from patients suffering from lung fibrosis (fibrotic sarcoidosis lung or hypersensitive pneumonitis). (**B**) Additional characteristics of senescent cells such as an increase in the number of lysosomes, secretory vesicles and mitochondria are shown by transmission electron microscopy in PanINs. Scale bar, 1μm.

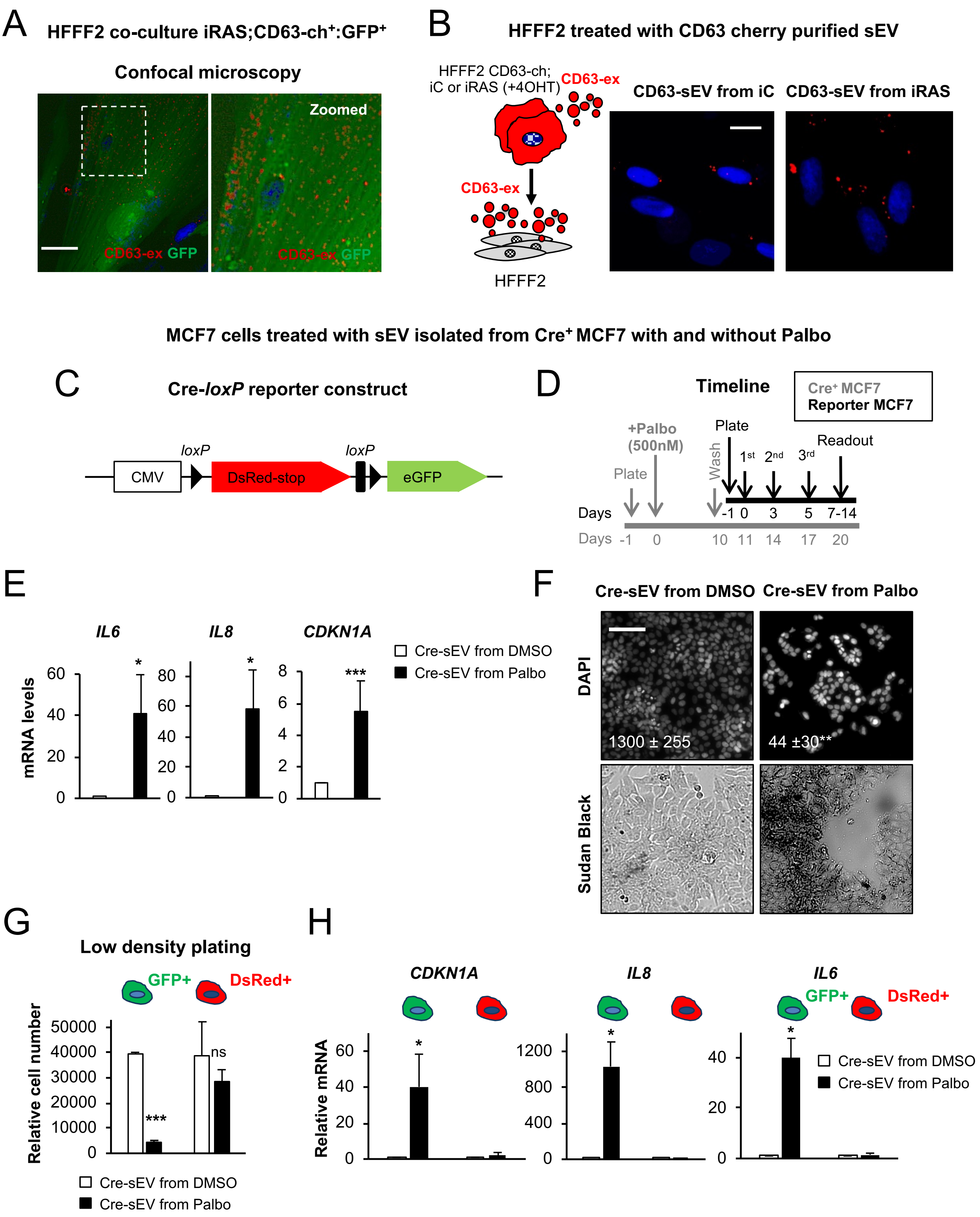
A
HFFF2 co-culture iRAS;CD63-ch+:GFP+
Confocal microscopy
Zoomed
CD63-ex GFP
CD63-ex GFP
B
HFFF2 treated with CD63 cherry purified sEV
HFFF2 CD63-ch; iC or iRAS (+4OHT)
CD63-ex
CD63-ex
HFFF2
CD63-sEV from iC
CD63-sEV from iRAS
MCF7 cells treated with sEV isolated from Cre+ MCF7 with and without Palbo
C
Cre-loxP reporter construct
CMV
loxP
DsRed-stop
loxP
eGFP
D
Timeline
Cre+ MCF7
Reporter MCF7
+Palbo (500nM)
Plate
Wash
Plate
1st
2nd
3rd
Readout
Days -1 0 3 5 7-14
Days -1 0 10 11 14 17 20
E
IL6
IL8
CDKN1A
mRNA levels
Cre-sEV from DMSO
Cre-sEV from Palbo
F
Cre-sEV from DMSO
Cre-sEV from Palbo
DAPI
1300 ± 255
44 ±30**
Sudan Black
G
Low density plating
GFP+
DsRed+
Relative cell number
ns
***
Cre-sEV from DMSO
Cre-sEV from Palbo
H
CDKN1A
IL8
IL6
GFP+
DsRed+
Relative mRNA
Cre-sEV from DMSO
Cre-sEV from Palbo

Figure S5. sEV derived from senescent cells induce senescence. Related to Figure 5
(**A**) Representative images showing the uptake of CD63-cherry positive sEV (CD63-sEV) in GFP^+ HFFF2 co-culture acquired with a confocal microscope. Scale bar, 30μm. (**B**) HFFF2 fibroblasts were treated for 72h with sEV isolated from iRAS;CD63-ch or HFFF2 cells expressing a vector control and mCherry-CD63 (iC;CD63-ch). Representative images show the uptake of CD63-sEV in HFFF2s treated with sEV isolated from both iC and iRAS. Scale bar, 20μm. (**C**) Scheme showing the *loxP* reporter construct expressed in the Reporter MCF7. (**D**) Diagram showing the timelines used to determine sEV uptake in Reporter MCF7. Briefly, Cre^+ MCF7 (grey font) were treated with 500nM Palbo for 10 days to establish senescence prior to the isolation of sEV. Reporter MCF7 (black font) were plated on day -1 of the start of the experiment and treated with freshly isolated Cre-sEV every 3 days for 1-2 weeks. (**E**) The establishment of senescence was determined by qPCR analysis. Data represent the mean ± SEM of 5 independent experiments (**F**) Reporter MCF7 treated with Cre-sEV isolated from Cre^+ MCF7 treated with Palbo activate senescence as shown by a decrease in proliferation (reduction in cell number counted by staining with DAPI) and an increase in cells staining positive for Sudan Black. Data represent the mean ± SD of 2-4 independent experiments. Scale bar, 100μm. (**G**) Low density proliferation assay and (**H**) qPCR quantification in GFP^+ and $DsRed^+$ sEV-treated cells sorted by FACS. Graphs show the mean ± SD of one of 2-4 biological experiments.

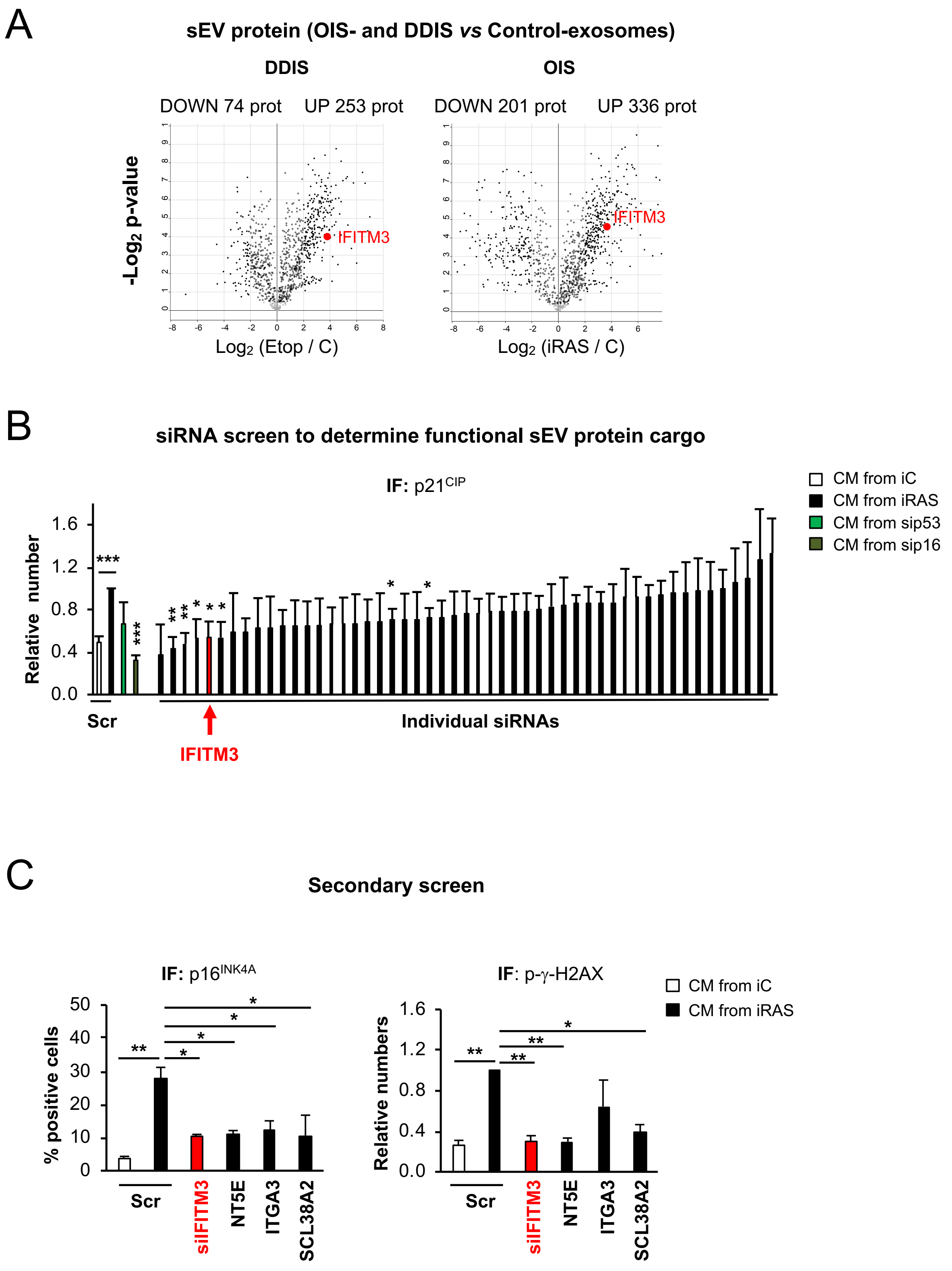
A
sEV protein (OIS- and DDIS vs Control-exosomes)
DDIS
OIS
DOWN 74 prot
UP 253 prot
DOWN 201 prot
UP 336 prot
-Log2 p-value
IFITM3
Log2 (Etop / C)
Log2 (iRAS / C)
B
siRNA screen to determine functional sEV protein cargo
IF: p21CIP
CM from iC
CM from iRAS
CM from sip53
CM from sip16
Relative number
1.6
1.2
0.8
0.4
0.0
Scr
Individual siRNAs
IFITM3
C
Secondary screen
IF: p16INK4A
% positive cells
50
40
30
20
10
0
Scr
siIFITM3
NT5E
ITGA3
SCL38A2
IF: p-γ-H2AX
Relative numbers
CM from iC
CM from iRAS

Figure S6. Further validation of proteins functionally implicated in inducing paracrine senescence mediated by sEV. Related to Figure 6
(**A**) Volcano plot showing proteins differentially expressed in sEV isolated from HFFF2s treated with Etop (DDIS; left panel) or iRAS HFFF2s (OIS; right panel). Both plots show that most proteins within sEV are upregulated during senescence compared to the controls (numbers indicate proteins significantly up or down-regulated). IFITM3 is highlighted in red. (**B**) HFFF2s treated with different CM of iRAS cells previously transfected with siRNA SMART pools were stained to determine the levels of $p21^{CIP}$ by IF. IFITM3 is highlighted in red. sip53 and sip16 were used as positive control for the screen (green bars). Data represent the mean ± SEM of 3-4 independent experiments. Stats represent statistical differences with the CM iRAS sample. (**C**) Secondary screen with the top 4 siRNA preventing the cell cycle arrest. We next evaluated additional markers of senescence by staining and quantifying CM-treated HFFF2s for $p16^{INK4A}$ and p-γH2AX. One-way ANOVA test was performed. Data represent the mean ± SEM of 2-3 independent experiments.

Borghesan *et al.* **Figure S7**

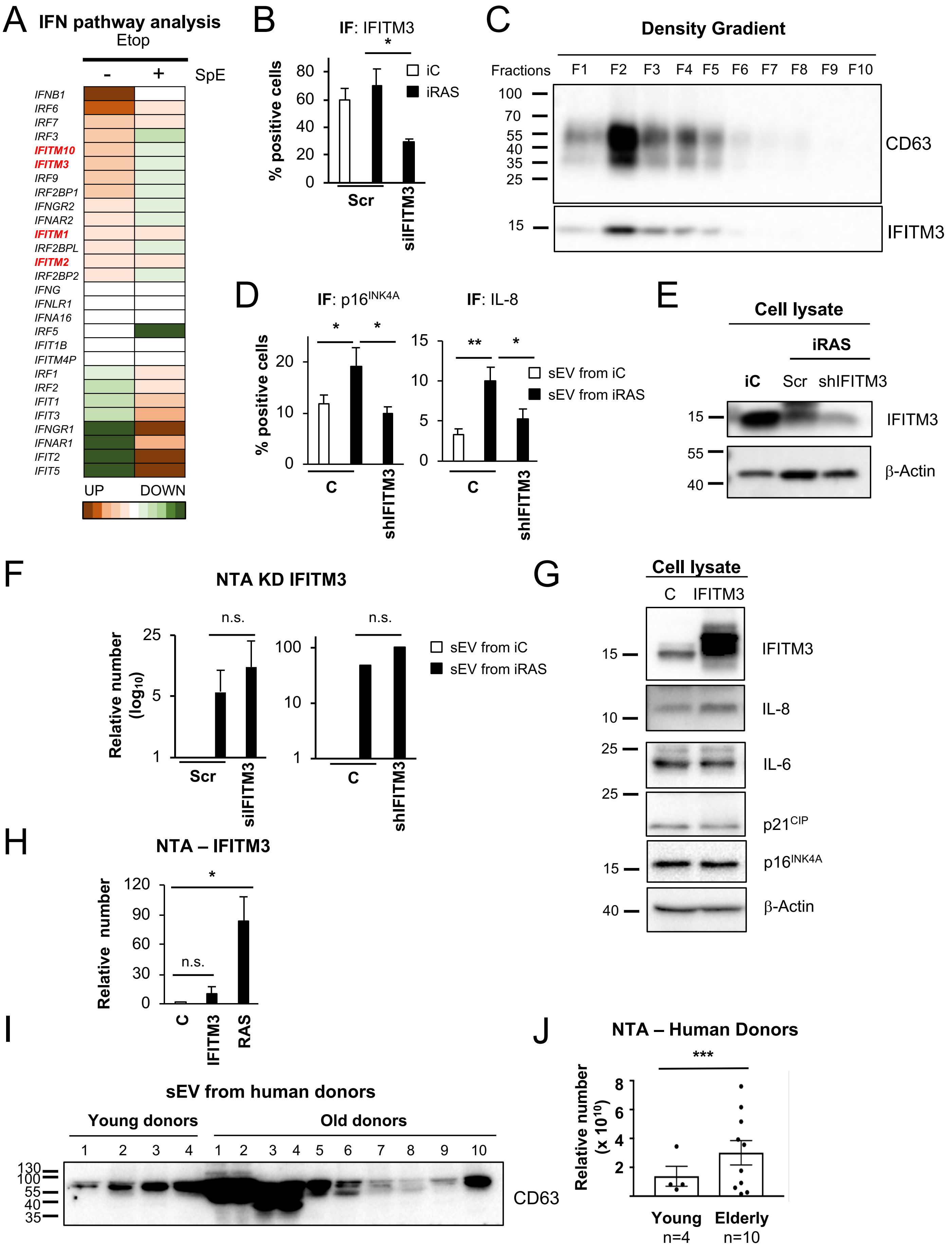

Figure S7. IFITM3 partially mediates paracrine senescence. Related to Figure 7
(**A**) HFFF2s incubated with sEV derived from HFFF2 undergoing DDIS (+Etop) show an increase in transcripts related to the interferon (IFN) pathway, in particular *IFITM* (in red) and *IFIT* mRNAs, which are downregulated when HFFF2s are incubated with sEV isolated from Etop+SpE treated cells. Data are normalized to the control. (**B**) IF showing the downregulation of IFITM3 in iC and iRAS cells transfected with a Scr or siIFITM3. Data represent the mean ± SEM of 3 independent experiments. (**C**) Immunoblotting for IFITM3 and CD63 in sEV derived from iRAS isolated by density gradient followed by ultracentrifugation. (**D**) Normal HFFF2 cells were treated with sEV derived from iRAS cells expressing a Control (C) or a previously validated shRNA targeting *IFITM3* (shIFITM3). The protein levels of $p16^{INK4A}$ and IL-8 were then determined by IF. Data show the mean ± SD of technical quadruplicates from 2 independent experiments. (**E**) Immunoblot for IFITM3 in iRAS cells expressing a shIFITM3 construct. β-Actin is used as loading control. (**F**) NTA analysis of sEV released in iRAS cells upon depletion of IFITM3 by siRNA or shRNA. (**G**) Immunoblotting analysis of HFFF2 ectopically expressing IFITM3 showing the levels of IFITM3 and different markers of senescence. (**H**) NTA analysis of particles released upon the ectopic expression of IFITM3 in HFFF2 cells. (**I**) Immunoblotting for CD63 in sEV isolated from the plasma of young and old donors. (**J**) NTA analysis of sEV derived from the plasma of young (n=4) and elderly (n=10) human donors. Chi-square analysis was performed.

Table S1. Proteins selected for the siRNA screen. Related to Figure 6

Protein IDs	Protein names	Gene names	Student's T-test	Mean Etopo_log	Student's T-test	Mean iRAS_log2(	Intensity CTR_01	Intensity CTR_01b	Intensity CTR_02	Intensity CTR_02b	Intensity Etopo_01	Intensity Etopo_01b	Intensity Etopo_02	Intensity Etopo_02b	Intensity iRAS_01	Intensity iRAS_01b	Intensity iRAS_02	Intensity iRAS_02b
P20591;P205	Interferon-induced GTP-bin	MX1	+	6.63	+	3.78	233470	40386	139490	114850	4798400	4935100	4054600	4167400	537160	617570	426310	264970
P61769	Beta-2-microglobulin;Beta-:	B2M	+	6.43	+	5.18	124560	127440	80328	92978	7639100	7895300	6708400	8528700	3448200	2337900	2617700	2195900
Q03135;P56	Caveolin-1	CAV1	+	5.74	+	4.56	49151	103420	33746	0	2021500	1295700	1551200	1688400	465000	473050	306880	406110
P23229	Integrin alpha-6;Integrin alp	ITGA6	+	5.61	+	7.76	211180	319890	27792	61478	1618000	1936100	1869300	1326900	10895000	10789000	9530400	10867000
Q7Z304	MAM domain-containing pr	MAMDC2	+	5.34	+	7.62	19048	7374.6	0	45420	1425300	1524100	1275200	1549900	6207700	6945800	5269800	6842400
P08648	Integrin alpha-5;Integrin alp	ITGA5	+	4.84	+	6.21	349040	419860	75568	85434	3546400	4298700	2904000	3864200	12766000	12321000	9432900	12491000
Q13219	Pappalysin-1	PAPPA	+	4.83	+	6.48	67456	46320	0	0	848890	1037100	730180	989190	19053000	2553000	13974000	23572000
P26022	Pentraxin-related protein P	PTX3	+	4.82	+	3.93	92444	247690	443770	476230	10781000	10414000	9497200	10244000	5259800	4344900	7068700	7611700
Q08380	Galectin-3-binding protein	LGALS3BP	+	4.45	+	4.78	1137300	1209500	941100	1274000	34641000	40083000	31308000	40091000	53339000	55489000	41003000	56693000
O14672	Disintegrin and metalloprot	ADAM10	+	4.32	+	6.94	81545	98826	53090	0	1493400	1287300	1157400	1325700	8726300	8627500	6972200	8447400
P26006	Integrin alpha-3;Integrin alp	ITGA3	+	4.32	+	5.20	723840	751670	194000	206140	11406000	13118000	9505100	11635000	24687000	21532000	19173000	24817000
Q6YHK3	CD109 antigen	CD109	+	4.19	+	5.84	136830	71788	65869	116890	1376500	1386400	1061600	1321200	3776900	4280000	3112500	3686900
Q04826;P30	HLA class I histocompatibilit	HLA-B	+	4.12	+	3.65	336360	161380	18993	82782	3413800	4043700	2638800	2679900	2629100	3037800	2209200	2254900
Q969P0	Immunoglobulin superfamil	IGSF8	+	4.08	+	5.49	156440	110970	20683	38001	1582800	1716300	1216900	1139400	3363100	4232300	2713500	4140400
P21589	5-nucleotidase	NT5E	+	4.00	+	5.02	1898800	1738100	302070	308900	24594000	26917000	22268000	23884000	56369000	60208000	42312000	47361000
Q9NVM1	Protein eva-1 homolog B	EVA1B	+	3.99	+	5.33	72437	0	18425	0	930680	841960	882930	635570	2117900	2674500	1938500	2362300
P15144	Aminopeptidase N	ANPEP	+	3.83	+	5.14	11206000	9343500	754540	779640	92321000	88562000	70822000	79793000	193180000	211470000	162370000	224690000
Q01628;P13	Interferon-induced transme	IFITM3	+	3.77	+	3.71	146580	119430	103410	30258	1395500	1464000	1249800	1583600	1491000	1892800	1082200	1540500
Q13641	Trophoblast glycoprotein	TPBG	+	3.75	+	4.70	107470	130150	0	21789	1535100	1568100	1212600	1446300	3532900	3048000	2733700	2740900
P12110	Collagen alpha-2(VI) chain	COL6A2	+	3.71	+	7.36	1718900	1349100	698410	568330	16553000	16674000	14455000	16668000	216790000	256070000	147360000	219970000
P13987	CD59 glycoprotein	CD59	+	3.68	+	4.52	634370	645330	0	141350	4738200	4736500	3383600	4764500	11277000	8955500	7836800	8510700
Q96QD8	Sodium-coupled neutral am	SLC38A2	+	3.68	+	3.70	152990	65516	59404	49596	1015700	1120600	1044300	1129300	1114300	1507800	871290	1192500
P13746;P04	HLA class I histocompatibilit	HLA-A	+	3.59	+	3.47	304030	361150	209400	230840	9061100	10425000	6988800	8517800	6735500	6852900	4805700	6559200
P13612	Integrin alpha-4	ITGA4	+	3.58	+	7.10	75368	85620	8667.9	10408	703120	679470	567620	1084000	4766600	4881000	3426000	4981200
P13726	Tissue factor	F3	+	3.58	+	4.63	58501	36109	16791	0	683260	644440	588150	432420	1163800	1533300	1439100	1515200
P00533;P04	Epidermal growth factor re	EGFR	+	3.57	+	5.19	126940	81221	19661	38267	730170	602410	711140	665950	2506600	2961400	1671600	2617100
P35613	Basigin	BSG	+	3.49	+	4.32	436970	602600	80999	60158	3562700	3425300	2541300	2935500	6630000	6484100	5369800	6057000
Q5ZPR3	CD276 antigen	CD276	+	3.43	+	4.17	55778	141470	0	0	827170	1188700	879100	926390	1913100	2094800	1323200	1819200
P12111	Collagen alpha-3(VI) chain	COL6A3	+	3.43	+	7.50	9461900	9079300	2928600	3043300	86364000	91872000	69335000	72739000	1469800000	1517400000	1028300000	1438000000
P21926	CD9 antigen	CD9	+	3.36	+	4.07	1215000	399080	51490	61369	4634100	5884700	3787600	5888600	11410000	9983600	2737200	13271000
P05556	Integrin beta-1	ITGB1	+	3.36	+	4.50	4595200	4339500	774270	1088100	32601000	32787000	26670000	33024000	60947000	67507000	49922000	67000000
O00560	Syntenin-1	SDCBP	+	3.36	+	4.50	1948100	1701300	213800	655410	10841000	11490000	10476000	11861000	24799000	28269000	21559000	26039000
Q7Z5G4	Golgin subfamily A member	GOLGA7	+	3.34	+	3.54	0	67266	0	0	257150	366600	222410	224510	309720	333310	243210	279380
P08473	Neprilysin	MME	+	3.29	+	4.94	1182300	1052200	237850	173340	6884400	7071400	6352000	6004700	22357000	22101000	15724000	19141000
P16070	CD44 antigen	CD44	+	3.25	+	3.65	1423400	1231400	215340	132300	10139000	11651000	8287700	10862000	18835000	17633000	12517000	13843000
P55290	Cadherin-13	CDH13	+	3.06	+	3.78	36653	54539	22423	0	662640	627700	611140	513460	1554200	1470800	1105900	1111600
P29317;Q15	Ephrin type-A receptor 2	EPHA2	+	3.02	+	3.81	379040	252670	97627	79944	1947300	2193200	1998500	2070700	3009900	3577000	2304300	2745500
Q8N474	Secreted frizzled-related pr	SFRP1	+	2.97	+	3.54	9104.4	52467	19928	0	608030	674330	564380	689960	1020600	1241500	664740	752720
P60033	CD81 antigen	CD81	+	2.86	+	3.68	1173500	1318000	722560	870050	8817300	8730400	7409600	8122300	17568000	16464000	17040000	22733000
P06756	Integrin alpha-V;Integrin alp	ITGAV	+	2.86	+	3.88	429490	415810	55717	8067.5	2017300	2180200	1545700	1404900	4719700	5228100	3366100	4295300
P27701	CD82 antigen	CD82	+	2.70	+	4.37	363200	300810	0	0	1460700	1398100	1064700	1167600	5010800	5062700	2421000	5087000
P24821	Tenascin	TNC	+	2.59	+	4.06	16157000	14072000	6600100	6379400	75831000	82657000	64564000	69995000	191790000	200270000	183550000	244020000
P17301	Integrin alpha-2	ITGA2	+	2.50	+	4.42	3243900	3215100	214940	227070	11909000	12566000	9423100	11750000	45193000	48867000	29818000	36853000
O75954	Tetraspanin-9	TSPAN9	+	2.45	+	4.90	391520	296180	42671	0	920300	1094100	976780	780260	4847200	6406700	4294500	5374300
P12109	Collagen alpha-1(VI) chain	COL6A1	+	2.35	+	5.86	4209100	3318700	2910000	4121500	22327000	23025000	19355000	22741000	235970000	259020000	181080000	231850000
Q9H5V8	CUB domain-containing pro	CDCP1	+	2.35	+	4.10	570610	608460	34527	36092	1940700	1598200	1826400	1817300	8879300	10339000	6762000	8061300
P05362	Intercellular adhesion mole	ICAM1	+	2.33	+	3.85	581730	583300	264160	233290	2322100	2554100	2013200	2393200	8503800	7815700	6818400	7919700
P10909	Clusterin;Clusterin beta cha	CLU	+	1.89	+	3.65	248810	222880	291820	273640	1882200	1966000	1547800	1779900	6443800	6589100	5501400	7307600
P50281;Q9Y	Matrix metalloproteinase-1	MMP14	+	1.73	+	4.43	385280	504420	19099	32453	1370400	1548100	1134100	1046600	5196000	5252600	3596400	4008900
Q6UVK1	Chondroitin sulfate proteog	CSPG4	+	1.57	+	3.59	1116200	998280	661970	611540	2863800	3362000	2113100	2984500	16034000	16746000	14335000	16952000
P62079	Tetraspanin-5	TSPAN5		1.38	+	3.61	0	0	0	0	36284	85813	29063	0	546290	512460	435910	381820

Table S2. Primers used for RT-PCR analysis. Related to STAR METHODS.

Target	Forward primer	Reverse primer
RPS14	CTGCGAGTGCTGTCAGAGG	TCACCGCCCTACACATCAAACT
CDKN2A	CGGTCGGAGGCCGATCCAG	GCGCCGTGGAGCAGCAGCAGCT
CDKN1A	CCTGTCACTGTCTTGTACCCT	GCGTTTGGAGTGGTAGAAATC
IL8	GAGTGGACCACACTGCGCCA	TCCACAACCCTCTGCACCCAGT
IL6	CCAGGAGCCCAGCTATGAAC	CCCAGGGAGAAGGCAACTG
IL1A	AGTGCTGCTGAAGGAGATGCCTGA	CCCCTGCCAAGCACACCCAGTA
IL1B	TGCACGCTCCGGGACTCACA	CATGGAGAACACCACTTGT
ITGB3	GGGGTAGGTTGGGAGAATGT	TCTGGGACAAAGGCTAAGGA
MMP3	AAGCTCTGAAAGTCTGGGAAGA	TCCCTGTTGTATCCTTTGTCCA
RAS	CCAGCTGATCCAGAACCATT	ATGGCAAACACACACAGGA
RPS8	GTTCAGCCACCCGAGATTGA	CCCATCACGAATGGGGTTCA
